# Emerging Trends in the Coordination Chemistry of Thiazolidinone-Containing Polydentate Ligands: Unconventional Binding Modes in Silver Complexes

**DOI:** 10.1021/acs.inorgchem.5c01815

**Published:** 2025-06-27

**Authors:** Julio Corredoira-Vázquez, Manuel Saa, Isabel García-Santos, Alfonso Castiñeiras, Matilde Fondo

**Affiliations:** † Departamento de Química Inorgánica, Facultade de Química, 16780 Universidade de Santiago de Compostela, Campus Vida, 15782 Santiago de Compostela, Spain; ‡ Institute of Materials (iMATUS), 16780 Universidade de Santiago de Compostela, 15782 Santiago de Compostela, Spain; § Departamento de Química Inorgánica, Facultade de Farmacia, Universidade de Santiago de Compostela, Campus Vida, 15782 Santiago de Compostela, Spain

## Abstract

This work presents an efficient and straightforward method for obtaining two previously described thiazolidinone-based ligands, HAm4DHotaz (*N′*-(4-oxothiazolidin-2-ylidene)­picolino-hydrazonamide) and Am4Eotaz (*N′*-(3-ethyl-4-oxothiazol-idin-2-ylidene)­picolinohydrazonamide), with high yield and purity. Moreover, the reactivity of these ligands toward different Ag^+^ salts has been explored, resulting in the isolation of four novel coordination complexes: two mononuclear species, [Ag­(HAm4DHotaz)_2_]­(NO_3_)·H_2_O (**1**·H_2_O) and [Ag­(Am4Eotaz)_2_]­(NO_3_) (**2**), and two tetranuclear compounds, [Ag_4_(Am4DHotaz)_4_]·8H_2_O (**3**·8H_2_O) and [Ag_4_(Am4Eotaz)_4_(NO_3_)_2_(H_2_O)]­(NO_3_)_2_·1.18H_2_O (**4**·1.18H_2_O). The crystal structures of **1**·H_2_O, **2**, **4**·1.18H_2_O, and **3**·3DMF, the latter obtained by recrystallization of [Ag_4_(Am4DHotaz)_4_]·8H_2_O in dimethylformamide, have been determined. Notably, in **4**·1.18H_2_O, the thiazolidinone moiety shows the unprecedented bridging mode μ_2_-κ^1^S:κ^1^S. In addition, **3**·3DMF represents a rare example of a thiazolidinone-containing complex featuring a μ_2_-κ^1^N:κ^1^S bridge. Moreover, across these complexes, the Am4Rotaz (R = DH or E) ligands display five hitherto unknown coordination modes. These ligands, and the auxiliary donors (present in **4**·1.18H_2_O), provide different Ag^+^ coordination environments: *AgN*_*4*_ cores with a seesaw geometry in the mononuclear complexes, and *AgN*_*2*_, *AgN*_*2*_ *S*, *AgN*_*4*_, *AgN*_*2*_ *OS*, *AgN*_*2*_ *S*_*3*_ or *AgN*_*2*_ *O*_*2*_ *S*_*3*_ cores with varied geometries in the tetranuclear ones. Furthermore, structures of the polynuclear species reveal significant argentophilic Ag···Ag interactions, which play a key role in directing the formation of the multinuclear frameworks.

## Graphical abstract

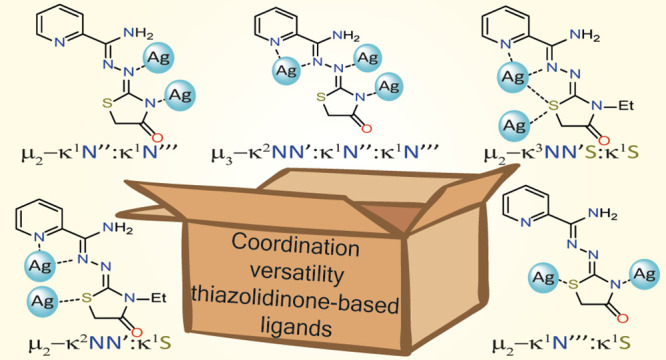

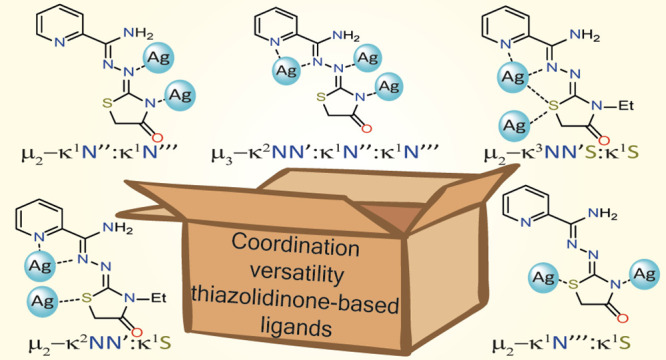

## Introduction

Thiazolidin-4-ones attract great interest due to their role as a structural core in numerous pharmaceutical agents. The biological activity of a broad spectrum of drugs is closely associated with the presence of these heterocycles in their molecular framework. Moreover, their pharmacological properties can be finely tuned and enhanced by functionalizing the basic thiazolidin-4-one scaffold, paving the way for the design and development of more effective and selective therapeutic agents.^1,2^

Despite the growing relevance these compounds have acquired in the field of medicine and their ongoing study for the development of future drugs,^3–5^ the coordination of these ligands to d-block metals, as well as the potential biological activity of these coordination compounds, remain areas that are still poorly explored. The ability of thiazolidinones to act as ligands, forming stable complexes with d-block metals, offers fertile ground for research. It is well-known that d-block metals possess unique properties that can be exploited to design coordination compounds with novel therapeutic properties. However, studies on the therapeutic activity of thiazolidinone complexes are hindered by the limited coordination chemistry developed for these small heterocycles. One of the main obstacles to advancing this chemistry appears to be the difficulty in obtaining these heterocycles in pure form.^2^ Besides, typically, isolating ligands that incorporate thiazolidinone residues requires complex, multistep synthetic procedures that are not only time-consuming but often involve the use of toxic reagents.^1,2^ As such, there is a clear need for the development of new, streamlined methods for the efficient synthesis of these donors, which would likely foster further progress in the coordination chemistry of these ligands.

In fact, it is surprising how few crystalline structures of metal complexes derived from thiazolidin-4-ones have been deposited in the Cambridge Structural Database (CSD).^6^ This is even more remarkable considering that the crystal structure of the first metal complex of this type was published almost half a century ago, in 1976.^7^ In actual, in addition to this complex, a literature search carried out in the CSD yields less than 20 articles on this topic,^8–24^ and it is restricted to the use of iron, copper, zinc, palladium, platinum, silver, mercury, thallium and tin as metals. These compounds show that the heterocycle typically acts as a terminal κ^1^N donor,^7,12,14,15,18–20,22,24^ though the coordination modes terminal κ^1^O,^10,21^ terminal κ^1^S,^8,13^ and bridge μ_2_-κ^1^N:κ^1^O^7,9,11^ have also been reported (Table 1, Schemes 1 and 2). Moreover, the μ_2_-κ^1^N:κ^1^S coordination mode has been recently introduced by us among the scarcely reported bridging behaviors of the thiazolidinone ring as ligand.^24^ In addition, some ligands incorporating this heterocycle have been described where the ring remains uncoordinated (Table S1).^15–20,22,23^

**Table 1:** Crystallographically Characterized Complexes with Thiazolidinone-Containing Ligands, Classified as a Function of the Coordination Mode of the Thiazolidinone Moiety

compound^a^	coordination mode^b^	ref
[Hg^II^Ph(DABRd)]	κ^1^N	^12^
[Hg^II^Me(TRd)]	κ^1^N	^12^
[(PhCH_2_)_2_Sn^IV^(Rd)(μ–OH)]_2_	κ^1^N	^14^
[*n*-Bu_2_Sn^IV^(Rd)(μ–OH)]_2_	κ^1^N	^14^
[Pd^II^(Am4DHotaz)Cl]	κ^1^N	^15^
[Pt^II^(Tone)Cl]	κ^1^N	^18,20^
[Cu^II^(Tone)Cl]	κ^1^N	^18^
[Fe^III^(NTone)_2_]Cl	κ^1^N	^19^
[Fe^III^(Tone)_2_](Fe^III^Cl_4_)	κ^1^N	^19^
[Cu^II^(Am4DHotaz)(H_2_O)_2_](ClO_4_)	κ^1^N	^22^
[Cu^II^(Am4DHotaz)(NO_3_)]	κ^1^N	^22^
[Tl^III^Me_2_(Rd)]	κ^1^S	^8^
[Pd^II^(L^1^)Cl_2_]	κ^1^S	^13^
[Sn^IV^Ph_3_Cl(L^2^)]	κ^1^O	^10^
[Sn^IV^Ph_3_Cl(L^3^)]	κ^1^O	^21^
[Tl^III^Me_2_(DABRd)]n^c^	μ_2_-κ^1^N:κ^1^O	^9^
[Tl^III^Me_2_(PyRd)]_2_ ^c^	μ_2_-κ^1^N:κ^1^O	^11^
[Tl^III^Me_2_(PyTd)]_2_ ^d^	μ_2_-κ^1^N:κ^1^O	^11^
[Ag^I^(L^4^)_2_(ClO_4_)]_2_	κ^1^N; μ_2_-κ^1^N:κ^1^O	^7^
[Ag^I^_8_(Am4DHotaz)_4_(NO_3_)_3_(H_2_O)(MeOH)](NO_3_)	κ^1^N; μ_2_-κ^1^N:κ^1^S	^24^
{[Ag^I^_8_(Am4DHotaz)_4_(NO_3_)_3_(H_2_O)_2_](NO_3_)}_n_	κ^1^N; μ_2_-κ^1^N:κ^1^S	^24^
{[Ag^I^_8_(Am4DHotaz)_4_(NO_3_)_2_(H_2_O)](NO_3_)(OH)}_n_	κ^1^N; μ_2_-κ^1^N:κ^1^S	^24^
{[Ag^I^_4_(Am4DHotaz)_4_]·3DMF}_n_	κ^1^N; μ_2_-κ^1^N:κ^1^S	this work
[Ag^I^_4_(Am4Eotaz)_4_(NO_3_)_2_(H_2_O)](NO_3_)_2_	κ^1^S; μ_2_-κ^1^N:κ^1^S; μ_2_-κ^1^S:κ^1^S	this work

a Solvates are omitted, ligands in Scheme 1.

b Coordination mode of the thiazolidinone ring, as represented in Scheme 2.

c Structure described as mononuclear but it is a polymer.

d Structure described as mononuclear but it is a dimer.

**Scheme 1:**
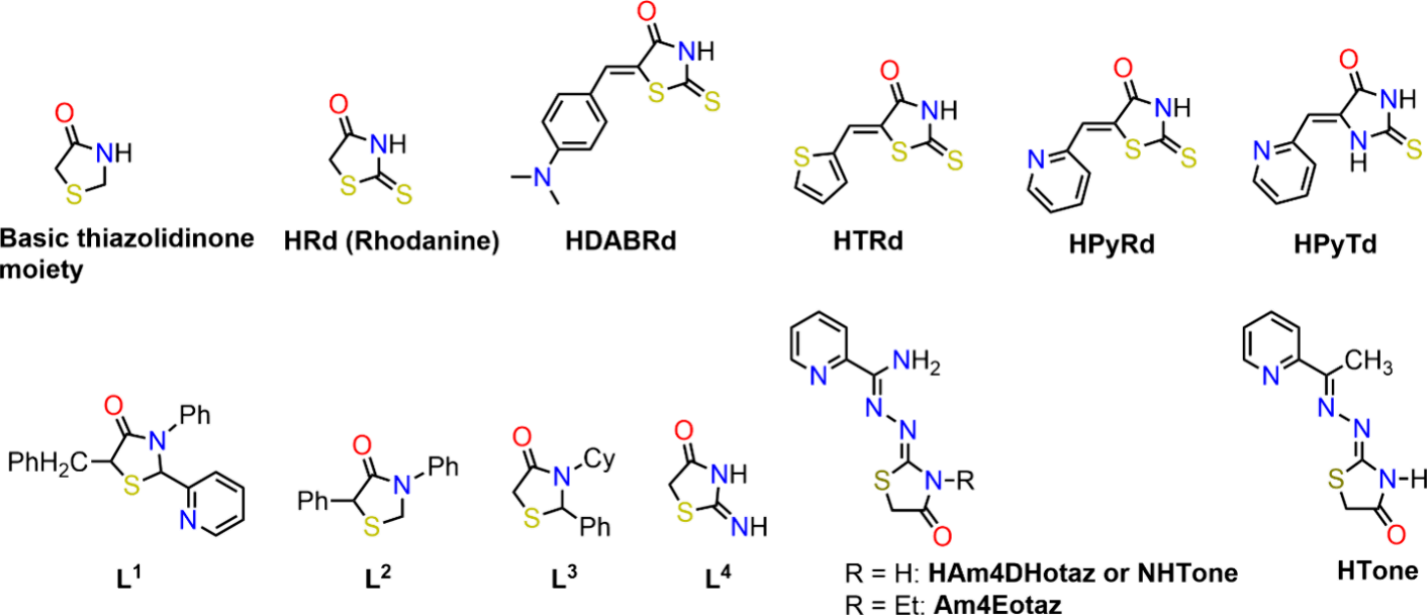
Ligands in the Complexes of Table 1

**Scheme 2:**
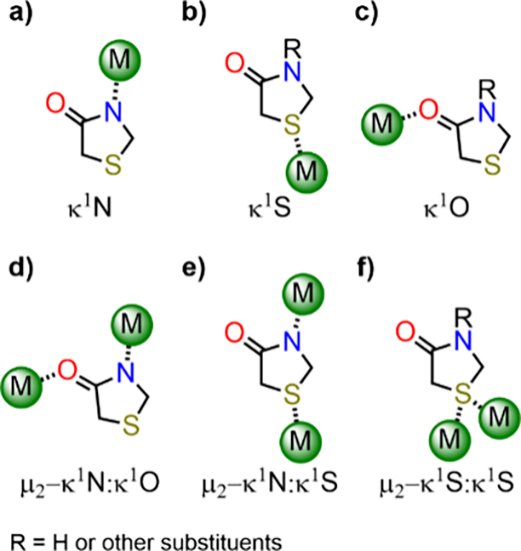
Coordination Modes of Thiazolidinones in Related Complexes (Table 1, a–e), and in the Complexes Described Herein (a,b,e,f).

Among these crystallographically characterized complexes, only a few have been evaluated for their cytostatic activity or their interaction with DNA.^15,18–20,22,23^ Nevertheless, some of these complexes have demonstrated enhanced in vitro activity compared to cisplatin.^19^ Therefore, this field seems to hold considerable promise for the development of novel therapeutic agents. However, as anticipated, its progress is currently limited by the scarcity of coordination chemistry studies involving these ligands.

In this context, the present work investigates the coordination behavior of two known thiazolidinone-based ligands (Scheme 3)^15,16,24^ toward silver. The findings reported in this study demonstrate that these ligands, along with their thiazolidinone heterocyclic moiety, exhibit a donor versatility beyond what was previously recognized. The resulting complexes broaden the spectrum of coordination modes for this small heterocycle, as well as for the Am4Rotaz (R = DH or E) donors, and may offer valuable insights into the mechanisms of action of future metal–thiazolidinone-based drugs.

**Scheme 3:**
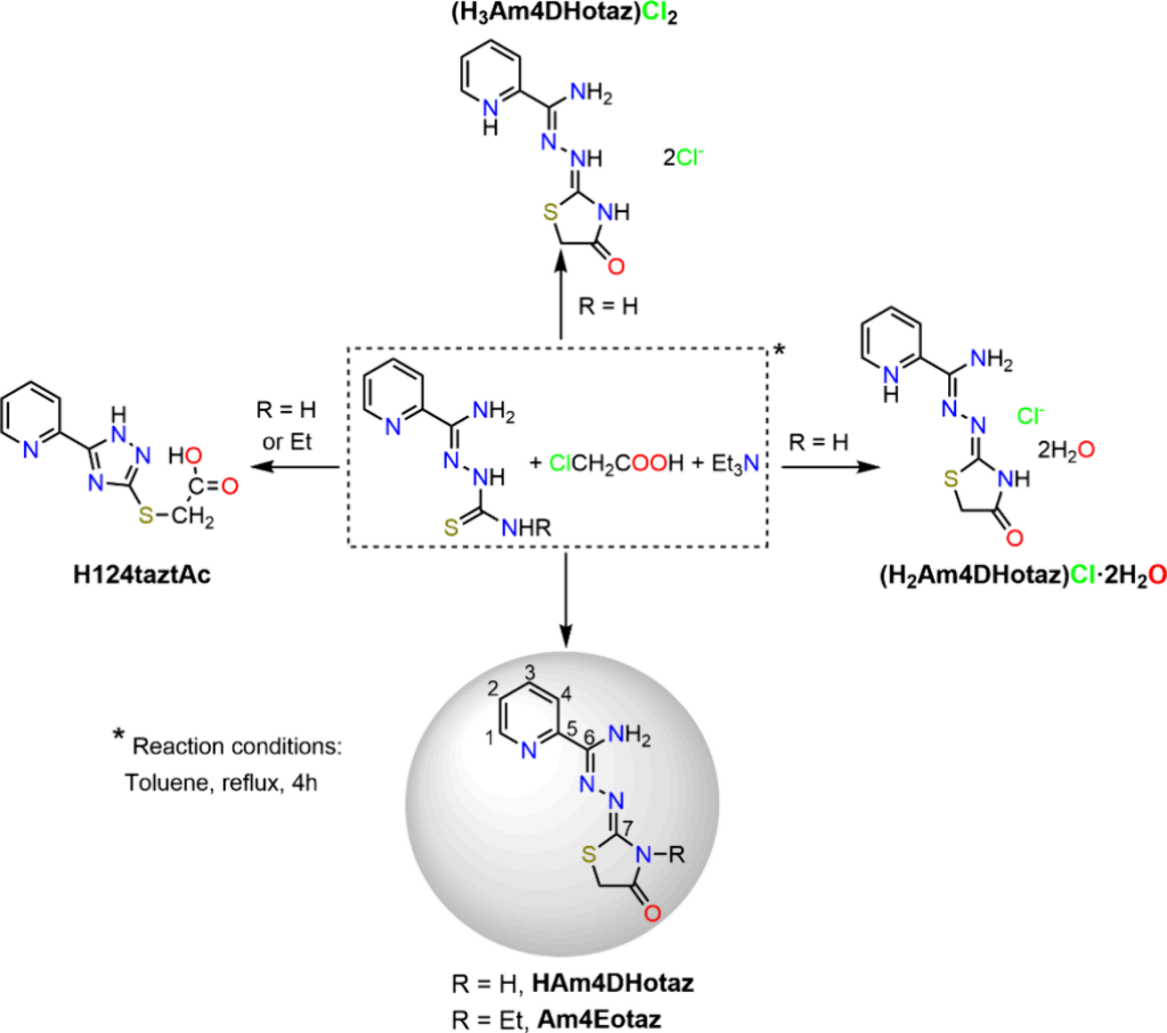
Reaction Scheme for Isolation of the Ligands (with Numbering Scheme for NMR), and the Side Products Identified in Their Syntheses

## Experimental Section

### Materials and General Methods

All chemical reagents were purchased from commercial sources, and used as received without further purification. Elemental analyses of C, H, and N were performed on a Themoscientific Flash Smart analyzer. Electrospray mass spectra of the complexes were recorded in methanol solutions in a mass spectrometer Bruker Microtof ESI-TOF. Infrared spectra were registered in the ATR mode on a BRUKER IFS-66v spectrophotometer in the range 4000–500 cm^–1^. The ^1^H and ^13^C NMR spectra were recorded on a BRUKER AMX 300 spectrometer.

### Synthesis of the Ligands

The ligands were synthesized using two different strategies: a conventional method and a microwave-assisted method. The synthesis by a *conventional procedure* was previously reported.^15,16^ Successive recrystallizations in methanol to obtain pure compounds resulted in the formation of the side-products H124taztAc (H_2_Am4DHotaz)­Cl·2H_2_O and (H_3_Am4DHotaz)­Cl_2_ before the thiazolidinones could be isolated as pure ligands. In this recrystallization processes, single crystals of Am4Eotaz were also isolated after 2 days. The crystal structures of the side products and Am4Eotaz were not reported, and they will be included here.

#### Microwave Assisted-Method

This procedure is the same for both ligands, and the method, very similar to a synthetic process previously described,^25^ is summarized below: a suspension of the corresponding thiosemicarbazone in Scheme 3 (8 mmol) in toluene (75 mL) was heated with magnetic stirring for 10 min. Then, a solution of chloroacetic acid (16 mmol) in toluene (25 mL) with triethylamine (1.5 mL) was added. The resulting solution was refluxed and subjected to 360 W microwave radiation for 1 h and 30 min. After this time, and once the reaction has cooled, the precipitate was filtered off and recrystallized in methanol, yielding the corresponding thiazolidin-4-one as a pure product.

#### HAm4DHotaz

Yield: 81%. Mp 205 °C. Anal. Calc. for C_9_H_9_N_5_OS: C, 45.95; H, 3.86; N, 29.77; S, 13.63%; found: C, 45.84; H, 3.95; N, 29.64; S, 13.92%. IR (ν∼/cm^–1^): 3438 ν­(NH), 1709 ν­(C=O), 1612–1524 ν­(C=N) + ν­(C=C). ^1^H NMR (DMSO-d_6_, ppm): 11.7 (1H, bs, NH), 8.62 (1H, s, H1), 8.10 (1H, d, H4), 7.90 (1H, t, H3), 7.45 (1H, t, H2), 6.45 (2H, bs, NH_2_), 3.82 (2H, s, CH_2_). ^13^C NMR (DMSO-d_6_, ppm): 174.09 (C=O), 161.64 (C7), 154.73 (C6), 149.46 (C5), 148.77 (C1), 138.14 (C3), 127.07 (C2), 122.60 (C4), 33.94 (CH_2_).

#### Am4Eotaz

Yield: 98%. Mp 168 °C. Anal. Calc. for C_11_H_13_N_5_OS: C, 46.45; H, 5.76; N, 23.50; S, 10.78%; found: C, 46.62; H, 5.56; N, 23.53; S, 10.67%. IR (ν∼/cm^–1^): 3458–3364 ν­(NH), 1706 ν­(C=O), 1612–1529 ν­(C=N) + ν­(C=C). ^1^H NMR (DMSO-d_6_, ppm): 8.61 (1H, s, H1), 8.12 (1H, d, H4), 7.88 (1H, t, H3), 7.48 (1H, s, H2), 6.63 (2H, bs, NH_2_), 3.88 (2H, s, CH_2_), 3.85 (2H, m, CH_2Et_), 1.17 (3H, t, CH_3Et_). ^13^C NMR (DMSO-d_6_, ppm): 172.11 (C=O), 156.27 (C7), 153.86 (C6), 150.68 (C5), 148.75 (C1), 137.24 (C3), 125.40 (C2), 121.07 (C4), 37.91 (CH_2_), 32.34 (CH_2Et_), 12.82 (CH_3Et_).

### Synthesis of the Complexes

All the complexes were obtained by a conventional method. In all cases, the solutions obtained and the solids isolated were protected from light with aluminum foil.

#### [Ag­(HAm4DHotaz)_2_]­(NO_3_)·H_2_O (**1**·H_2_O)

A solution of AgNO_3_ (34.0 mg, 0.2 mmol) in H_2_O (3 mL) was added to a solution of HAm4DHotaz (47.0 mg, 0.2 mmol) in MeOH (20 mL), and the mixture was stirred for 15 min. The resulting light-yellow solution was kept in the dark, and slow evaporation of the mother liquors for 4 days yielded colorless crystals of **1**·H_2_O, suitable for X-ray diffraction analysis. Yield: 30%. Mp 205 °C. Anal. Calc. for C_18_H_20_AgN_11_O_6_S_2_: C, 32.8; H, 3.1; N, 23.4; S, 9.7; found: C, 33.0; H, 2.9; N, 23.5; S, 9.5%. Mass spectra (ESI^+^, *m*/*z*): 342.0 [Ag­(HAm4DHotaz)]^+^, 577.0 [Ag­(HAm4DHotaz)_2_]^+^. IR (ν ~/cm^–1^): 3522 ν­(OH), 3462–3311 ν­(NH), 1710 ν­(C=O), 1589, 1563 ν­(C=N) + ν­(C=C), 1384 ν­(NO_3_^–^), 1003 ν­(NN). RMN ^1^H (DMSO-d_6_, ppm): 11.7 (1H, s, NH); 8.58 (1H, d, H1); 8.09 (1H, d, H4); 8.0 (1H, t, H3); 7.95 (1H, t, H2); 6.70 (2H, bs, NH_2_), 3.85 (2H, s, CH_2_).

The same compound is isolated when AgNO_3_ and HAm4DHotaz are mixed in 1:2 molar ratios.

#### [Ag­(Am4Eotaz)_2_]­(NO_3_) (**2**)

A solution of AgNO_3_ (34 mg, 0.2 mmol) in H_2_O (3 mL) was added to a solution of Am4Eotaz (105.3 mg, 0.40 mmol) in MeOH (20 mL) and stirred for 15 min. The pale-yellow solution was kept in the dark, and slow evaporation of the mother liquors over the course of 1 week led to the formation of colorless single crystals of **2**. Yield: 53%. Mp 215 °C. Anal. Calc. for C_22_H_26_AgN_11_O_5_S_2_: C, 37.9; H, 3.8; N, 22.1; S, 9.2; found: C, 37.7; H, 4.0; N, 21.9; S, 8.9%. Mass spectra (ESI^+^, *m*/*z*,): 370.0 [Ag­(Am4Eotaz)]^+^, 635.1 [Ag­(Am4Eotaz)_2_)]^+^. IR (ν ~/cm^–1^): 3578 ν­(OH), 3488–3367 ν­(NH), 1708 ν­(C=O), 1530–1585 ν­(C=N) + ν­(C=C), 1384 ν­(NO_3_^–^), 995 ν­(NN). ^1^H NMR (DMSO-d_6_, ppm): 8.61 (1H, d, H1); 8.13 (1H, d, H4); 7.89 (1H, t, H3); 7.47 (1H, t, H2); 6.65 (2H, bs, NH_2_); 3.88 (2H, s, CH_2_); 3.83 (2H, c, CH_2Et_); 1.17 (3H, t, CH_3Et_).

#### [Ag_4_(Am4DHotaz)_4_]·8H_2_O (**3**·8H_2_O)

A solution of HAm4DHotaz (20 mg, 0.098 mmol) in MeOH (25 mL) was stirred while adding AgOAc (16.4 mg, 0.098 mmol). The resulting mixture was stirred for 10 min more. Slow evaporation of the mother waters in the dark precipitated **3**·8H_2_O as a microcrystalline material. Yield: 90%. Mp 220 °C. Anal. Calc. for C_36_H_48_Ag_4_N_20_O_12_S_4_: C, 28.6; H, 3.2; N, 18.5; S, 8.5%; found: C, 28.5; H, 3.2; N, 18.2; S, 8.6%. Mass spectra (ESI^+^, *m*/*z*): 684.9 [Ag_2_(Am4DHotaz)_2_+H]^+^, 792.8 [Ag_3_(Am4DHotaz)_2_]^+^, 1133.8 [Ag_4_(Am4DHotaz)_3_]^+^. IR (ν ~/cm^–1^): 3574 ν­(OH), 3484–3358 ν­(NH), 1703 ν­(C=O), 1520–1587 ν­(C=N) + ν­(C=C), 999 ν­(NN). ^1^H NMR (DMSO-d_6_, ppm): 8.55 (1H, d, H1), 8.04 (1H, d, H4), 7.84 (1H, t, H3), 7.42 (1H, t, H2), 6.76 (2H, bs, NH_2_), 3.74 (2H, s, CH_2_).

Recrystallization of the solid in DMF with a few drops of diethyl ether led to colorless single crystals of [Ag_4_(Am4DHotaz)_4_]·3DMF (**3**·3DMF) after 2 weeks, suitable for X-ray diffraction studies.

#### [Ag_4_(Am4Eotaz)_4_(NO_3_)_2_(H_2_O)]­(NO_3_)_2_·1.18H_2_O (**4**·1.18H_2_O)

A solution of AgNO_3_ (128.3 mg, 0.76 mmol) in H_2_O (3 mL) was added to a solution of Am4Eotaz (50.0 mg, 0.19 mmol) in MeOH (20 mL), and the mixture was stirred for 15 min. The resulting solution was protected from light, and after 1 week of slow evaporation, colorless crystals of **4**·1.18H_2_O, suitable for single X-ray diffraction analysis, were isolated. Yield: 35% based on the ligand. Mp 165 °C. Anal. Calc. for C_44_H_56.37_Ag_4_N_24_O_18.18_S_4_: C, 19.0; H, 3.2; N, 19.0; S, 7.2%; found: C, 18.8; H, 3.1; N, 18.8; S, 7.1%. Mass spectra (ESI^+^, *m*/*z*): 370.0 [Ag­(Am4Eotaz)]^+^, 635.1 [Ag­(Am4Eotaz)_2_)]^+^. IR (ν ~/cm^–1^): 3487, 3367 ν­(NH), 1708 ν­(C=O), 1528–1584 ν­(C=N) + ν­(C=C), 1384 ν­(NO_3_^–^), 995 ν­(NN). ^1^H NMR (DMSO-d_6_, ppm): 8.65 (1H, d, H1), 8.14 (1H, d, H4), 7.72 (1H, m, H3), 7.49 (1H, m, H2), 7.00–7.30 (2H, bs, NH_2_), 4.04 (2H, s, CH_2_), 3.88 (2H, c, CH_2Et_), 1.15 (3H, t, CH_3Et_).

### Single X-ray Diffraction Studies

Diffraction data for H124taztAc, (H_2_Am4DHotaz)­Cl·2H_2_O, (H_3_Am4DHotaz)­Cl_2_, Am4Eotaz, [Ag­(HAm4DHotaz)_2_]­(NO_3_)·H_2_O (**1**·H_2_O), [Ag­(Am4Eotaz)_2_]­(NO_3_) (**2**), [Ag_4_(Am4DHotaz)_4_]·3DMF (**3**·3DMF) and [Ag_4_(Am4Eotaz)_4_(NO_3_)_2_(H_2_O)]­(NO_3_)_2_·1.18H_2_O (**4**·1.18H_2_O) were recorded at 293(2) K (H124taztAc) or 100.0(1) K on a Bruker X8 KappaAPEXII diffractometer. Graphite monochromated MoK­(α) radiation (λ = 0.71073 A) was used throughout. The data were processed with APEX2 ^26^ and corrected for absorption using SADABS.^27^ The structures were solved by direct methods^28^ and refined by full-matrix least-squares techniques against *F*2.^29^ In complex **2**, there is a disordered region in the cell (volume ca. 74 Å^3^) which could not be satisfactorily described. Therefore, a correction by SQUEEZE^30^ was used. Positional and anisotropic atomic displacement parameters were refined for all non-hydrogen atoms. H-atoms were located in Fourier maps or placed in geometrically idealized positions. All H-atoms were refined using a riding model. Molecular graphics were generated with DIAMOND.^31^ Details on experimental and refinement results are summarized in Tables S2 and S3.

## Results and Discussion

### Synthesis

The conventional synthesis of HAm4DHotaz and Am4Eotaz produces side products (Scheme 3) that complicate their purification, and lead to yields below 70%.^15,16^ These byproducts, including H124taztAc and chloride salts of the protonated ligands (Scheme 3), were identified by X-ray diffraction. To improve the process, a microwave-assisted synthesis, which is postulated as an alternative approach for increasing the efficiency of synthesis of organic compounds,^32^ was developed. This approach, like a previously reported method,^25^ mixed the same reactants in toluene under reflux for 90 min. This significantly reduced reaction time (from 4 h to 90 min) and prevented side product formation, yielding purer ligands with improved yields (81% for HAm4DHotaz and 98% for Am4Eotaz). These were characterized by elemental analysis, IR and ^1^H and ^13^C NMR spectroscopy, and the results agree with those reported.^15,16^ In addition, single crystals of Am4Eotaz and the side products H124taztAc, (H_2_Am4DHotaz)­Cl·2H_2_O and (H_3_Am4DHotaz)­Cl_2_ could be solved.

Reaction of HAm4DHotaz or Am4Eotaz with AgOAc or AgNO_3_ in different molar ratios was studied, as summarized in Scheme 4. Thus, the reaction of the ligands with AgNO_3_ in 1:1 or 1:0.5 molar ratios in H_2_O/MeOH leads to the isolation of mononuclear complexes [AgL_2_]­(NO_3_)·nH_2_O as single crystals, independently of the thiazolidinone employed. In this case, the nitrogen atom of the HAm4DHotaz donor remains protonated. Accordingly, both ligands act as neutral donors.

**Scheme 4:**
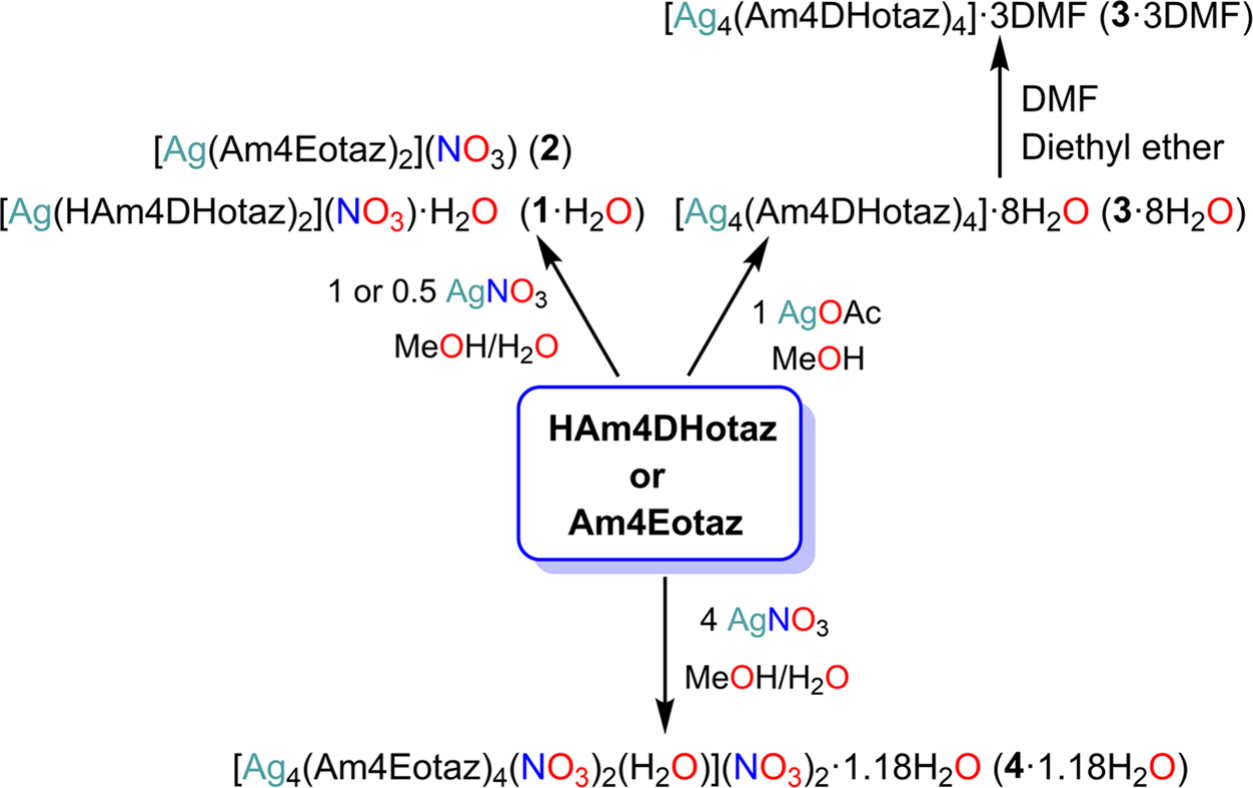
Reaction Scheme for Isolation of the Silver Complexes

However, when the same syntheses are repeated using AgOAc instead of AgNO_3_, the results obtained are quite different. In this case, HAm4DHotaz undergoes deprotonation to form the complex [Ag_4_(Am4DHotaz)_4_]·8H_2_O (**3**·8H_2_O), whose recrystallization in DMF renders single crystals of [Ag_4_(Am4DHotaz)_4_]·3DMF (**3**·3DMF), suitable for X-ray diffraction studies.

On the other hand, the product isolated from the reaction with Am4Eotaz under similar conditions could not be unequivocally characterized. However, when Am4Eotaz is reacted with AgNO_3_ in a 1:4 molar ratio, a tetranuclear complex is also obtained, rendering [Ag_4_(Am4Eotaz)_4_(NO_3_)_2_(H_2_O)]­(NO_3_)_2_·1.18H_2_O (**4**·1.18H_2_O) as single crystals. The same reaction with HAm4DHotaz generates a solid that could not be unequivocally identified. Nonetheless, we have previously shown that molar relations HAm4DHotaz:Ag 1:2 led to octanuclear complexes, where the molar ratio of the reactants is maintained in the isolated *Ag*_8_ compounds.

These observations suggest that complexes of analogous stoichiometry are only obtained when both thiazolidinone ligands act as neutral donors. For this reason, it appears that the silver salt has a profound impact on the nuclearity of the complexes, given that basic salts can deprotonate HAm4DHotaz. Furthermore, the study of these reactions reveals that ligand-to-silver molar ratios higher than 1:1 also influence the nuclearity of the resulting complexes. In addition, the findings clearly indicate that the stoichiometry of the final compounds does not necessarily correspond to the ratio of the starting reagents.

The four complexes were fully characterized by elemental analysis, mass spectrometry, IR and ^1^H NMR spectroscopy, and by X-ray diffraction studies. Mass spectrometry (Figure S1) allows identifying [Ag­(Am4Rotaz)_2_H]^+^ (R = DH or E) peaks for **1**·H_2_O and **2**, in agreement with the 1:2 Ag:L stoichiometry. Nevertheless, in the case of the tetranuclear complexes, fragments containing four Ag^+^ ions were observed for **3**·8H_2_O but not for **4**·1.18H_2_O, which could indicate that weaker bridges connect the Ag centers in **4**·1.18H_2_O.

### Spectroscopic Studies

The IR spectra of the complexes (Figure S2) display bands corresponding to ν­(NH) vibrations, in the 3500–3100 cm^–1^ range^33^ for the compounds derived from HAm4DHotaz, and between 3500 and 3300 cm^–1^ for those derived from Am4Eotaz, which are shifted to lower frequencies compared to the free ligands.^15,16^ In addition, the spectra of all the complexes, except for [Ag­(Am4Eotaz)_2_]­(NO_3_), show a broad around 3550 cm^–1^, assigned to the ν­(OH) vibration of H_2_O, either as hydrate or ligand. The bands associated with the ν­(C=N) and ν­(C=C) vibrations of the thiosemicarbazone backbone and the thiazolidinone ring appear in the 1520–1589 cm^–1^ range. These bands are slightly shifted to lower frequencies compared to the free ligands, which is consistent with the coordination of the imine N-atom. The strong band observed in all the spectra around 1707 cm^–1^ corresponds to the ν­(C=O) vibration. This band appears at a frequency similar to that of the free ligand, indicating that coordination does not occur through this donor group.

Moreover, a band at 1384 cm^–1^ in the IR spectra of [Ag­(HAm4DHotaz)_2_]­(NO_3_)·H_2_O, [Ag­(Am4Eotaz)_2_]­(NO_3_) and [Ag_4_(Am4Eotaz)_4_(NO_3_)_2_(H_2_O)]­(NO_3_)_2_·1.18H_2_O, which is absent in the spectrum of [Ag_4_(Am4DHotaz)_4_]·8H_2_O, supports the presence of NO_3_^–^ in these complexes.^24^

The ^1^H NMR spectra of all the complexes (Figure S3) show a unique set of signals, in agreement with chemical homogeneity of the samples and the apparent absence of side-products. As key highlights of these spectra, it can be noted that

1. The spectrum of [Ag­(HAm4DHotaz)_2_]·H_2_O shows a signal at 11.7 ppm, corresponding to the NH proton of the thiazolidinone ring. In contrast, the absence of this peak in the spectrum of [Ag_4_(Am4DHotaz)_4_]·8H_2_O is consistent with the ligands being in their deprotonated form in this tetranuclear compound.
2. The NH_2_ group in the complexes derived from HAm4DHotaz is at *ca*. 6.70 ppm but in [Ag_4_(Am4Eotaz)_4_(NO_3_)_2_(H_2_O)]­(NO_3_)_2_·1.18H_2_O, this signal appears more deshielded (7.0–7.3 ppm). In all these cases the signals are shifted downfield compared to the free ligands. This agrees with the delocalization of charge of the ligand, and the effect of the coordination upon conjugation.
3. The signals corresponding to the pyridine ring appear in all cases at 7–9 ppm, within the usual range for these aromatic protons, while the CH_2_ group of the thiazolidinone ring is located at 3.8–4.0 ppm, undergoing minimal shift compared to the free ligand.^15,16^

4. Complexes derived from Am4Eotaz exhibit peaks around 3.85 and 1.15 ppm, corresponding to the CH_2_ and CH_3_ groups of the ethyl group attached to the nitrogen atom of the ring.

The spectra of all the compounds also demonstrate the relative stability of the complexes in DMSO-*d*_6_ solution for a short period under light exposure (*ca*. 3 h), as no signs of decomposition are observed in these spectra.

### X-ray Diffraction Studies

The side products H124taztAc, (H_2_Am4DHotaz)­Cl·2H_2_O and (H_3_Am4DHotaz)­Cl_2_, the ligand Am4Eotaz, and the silver complexes **1**·H_2_O-**4**·1.18H_2_O were unequivocally characterized by single X-ray diffraction studies. The experimental data for their X-ray diffraction analyses are summarized in Tables S2 and S3. Main bond distances and angles for the four side products and Am4AEotaz, including hydrogen bond schemes, are summarized in Tables S4–S6, and their structures are shown in Figures S4 and S5.

#### [Ag­(HAm4DHotaz)_2_]­(NO_3_)·H_2_O (**1**·H_2_O) and [Ag­(Am4Eotaz)_2_]­(NO_3_) (**2**)

The crystal structures of these complexes are very similar and they will be discussed together. Their main bond distances and angles are recorded in Table 2 and their structures are shown in Figures 1 and S6.

**Figure 1:**
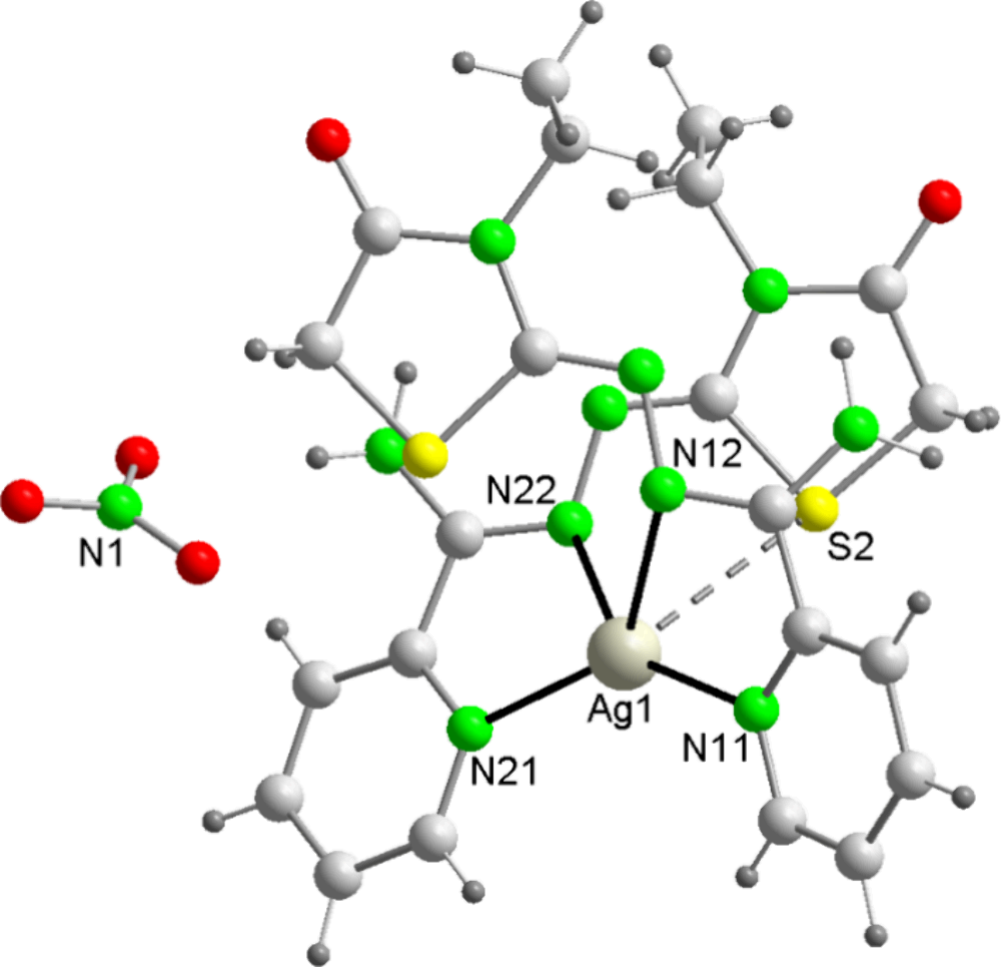
Molecular structure for [Ag­(Am4Eotaz)_2_]­(NO_3_) (**2**).

**Table 2:** Main Distances (Å) and Angles (°) for **1**·H_2_O and **2**

	1·H_2_O	2
Ag1–N11	2.252(2)	2.2077(17)
Ag1–N12	2.409(2)	2.6818(17)
Ag1–N21	2.235(2)	2.2778(17)
Ag1–N22	2.479(2)	2.3805(17)
N21–Ag1–N11	140.50(8)	138.43(6)
N11–Ag1–N22	138.53(8)	147.91(6)
N11–Ag1–N12	71.16(8)	69.37(6)
N12–Ag1–N22	96.59(8)	94.99(5)
N21–Ag1–N12	142.61(8)	137.39(6
N21–Ag1–N22	70.96(8)	71.78(6)

Both mononuclear complexes are ionic, containing [Ag­(L)_2_]^+^ cations and nitrate anions. In addition, water solvate is present in the unit cell of **1**·H_2_O. In the [Ag­(L)_2_]^+^ cations, the ligands act as neutral bidentate chelate donors, using their pyridine and imine nitrogen atoms (κ^2^NN′ coordination mode in Scheme 5), with the thiazolidinone ring remaining uncoordinated. This is the usual coordination mode of this kind of ligand when the N-atom of the thiazolidinone ring is substituted or remains protonated.^15,16,22,23^ Accordingly, this leads to an *AgN*_4_ core, with highly distorted seesaw geometry according to SHAPE (Table S7).^34^ This distortion is highlighted by the angles (Table 2), which are quite far from the ideal ones (90°, 120°, and 180°) in both complexes. However, these angles and the Ag–N distances are within the range of those previously reported for other Ag^+^ complexes with this geometry,^24,35^ and do not merit further consideration.

**Scheme 5:**
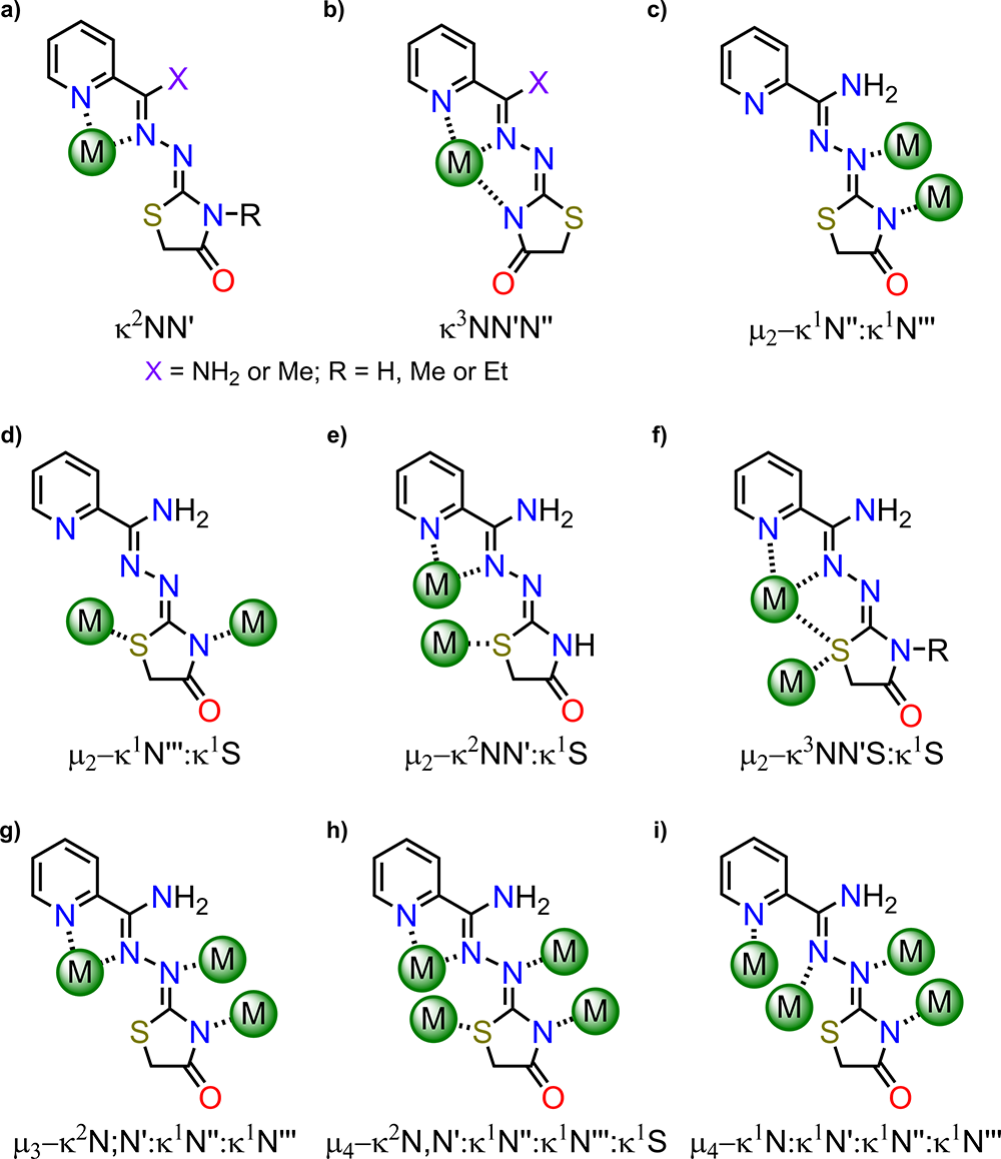
Coordination Modes for Am4Rotaz or Rtone Ligands (Scheme 1) in Metal Complexes Crystallographically Characterized (Table 4,a,b,h,i) and in Complexes Described Herein (a,c–g)

#### [Ag_4_(Am4DHotaz)_4_]·3DMF (**3**·3DMF)

The unit cell of this compound shows neutral tetranuclear [Ag_4_(Am_4_DHotaz)_4_] molecules (Figure 2) and DMF as solvate. Its main bond distances and angles are listed in Table 3.

**Figure 2:**
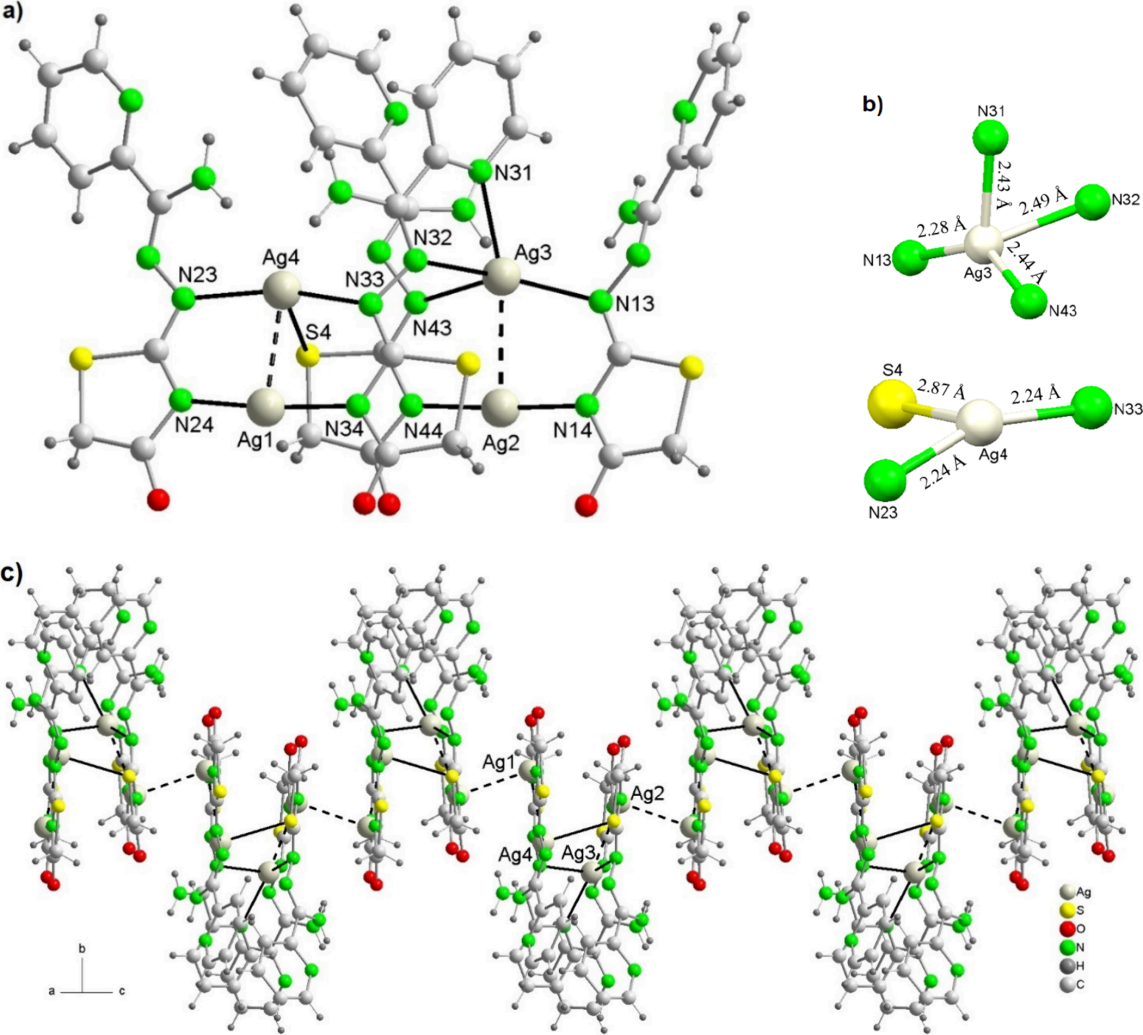
(a) Tetranuclear unit in **3**·DMF; (b) coordination environments of Ag3 and Ag4, with bond distances rounded to two decimal places; (c) 1D chain by argentophilic interactions.

**Table 3:** Main Distances (Å) and Angles (°) for **3**·3DMF

Ag1–N24	2.098(9)	Ag3–N13	2.286(9)	Ag4–N23	2.240(8)
Ag1–N34	2.097(9)	Ag3–N43	2.444(8)	Ag4–N33	2.240(8)
Ag2–N44	2.073(8)	Ag3–N31	2.425(9)	Ag4–S4	2.867(3)
Ag2–N14	2.081(8)	Ag3–N32	2.485(9)		
Ag1···Ag2	5.682(4)	Ag2···Ag3	2.9553(11)	Ag1···Ag4	2.8625(12)
Ag1···Ag2^a^	3.1682(11)	Ag3···Ag4	4.927(1)		
N24–Ag1–N34	165.9(3)	N13–Ag3–N31	113.7(3)	N23–Ag4–N33	158.4(3)
N44–Ag2–N14	173.6(3)	N13–Ag3–N43	139.2(3)	N23–Ag4–S4	90.0(2)
		N31–Ag3–N43	100.5(3)	N33–Ag4–S4	98.2(2)
		N13–Ag3–N32	134.9(3)		
		N31–Ag3–N32	66.4(3)		

a *x*, −*y* + 1/2, *z* – 1/2.

In this complex, the four metal centers are crystallographically distinct, with Ag···Ag distances between adjacent atoms ranging from 2.8626(12) to 5.682(4) Å. Thus, the Ag^+^ ions are located at the vertices of a trapezoid. In addition, some of these Ag^+^ ions are positioned at relatively short distances from Ag^+^ ions in adjacent molecules (Ag1···Ag2^1^ = 3.1682(11) Å, Table 3). Accordingly, some of these distances in the complex suggest the presence of significant Ag···Ag interactions. These are known as closed-shell argentophilic interactions (d^10^–d^10^), which arise from the overlap of filled 5d orbitals with empty 6s and 6p orbitals, and are therefore best described as strong van der Waals attractions. The presence and significance of these interactions in Ag^+^ complexes are assessed by comparison with van der Waals radii in pure silver. In metallic silver, the interatomic distance is 2.88 Å, while the sum of the van der Waals radii of two Ag atoms is 3.44 Å. In a complex, Ag···Ag distances <3.0 Å indicate significant argentophilic interactions, while separations >3.3 Å are considered weak and thus negligible. Argentophilic interactions may be supported by auxiliary ligands, or may be unsupported; the latter are indicative of a true bond.^36^ Accordingly, strong argentophilic interactions are observed between Ag1···Ag4 and Ag2···Ag3, with distances <3 Å (Table 3), but since these interactions are supported by auxiliary bridging ligands, they are not considered true bonds. The only unsupported interaction is between Ag1 and an Ag2 atom from a neighboring unit (*x*, −*y* + 1/2, *z* – 1/2), but since this distance is greater than 3.0 Å (Table 3), it is not considered a true Ag–Ag bond. Despite this, these latter van der Waals interactions link the *Ag*_4_ units into a chain, thus resembling a linear polymer (Figure 3c), showing that this kind of interaction may have a considerable influence on the orientation of coordination complexes in the crystalline state.^37^ The remaining interactions between Ag^+^ ions of neighboring molecules are all greater than 4 Å, making them very weak and not worth further discussion.

**Figure 3:**
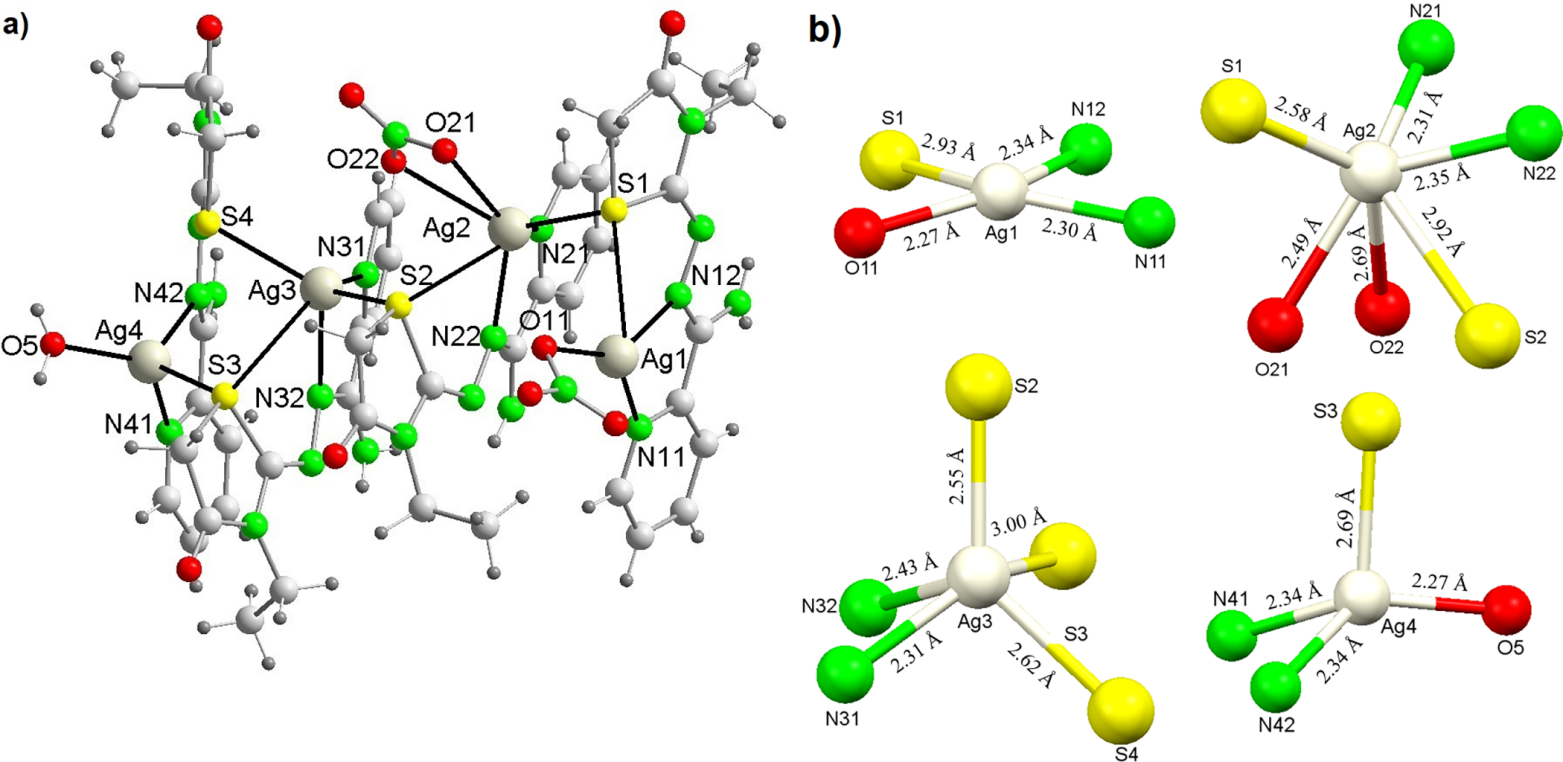
(a) Structure for the cation [Ag_4_(Am4Eotaz)_4_(NO_3_)_2_(H_2_O)]^2+^ in **4**·1.18H_2_O; (b) coordination environments of Ag centers, with bond distances rounded to two decimal places.

The coordination numbers of the Ag^+^ ions are different. Thus, Ag1 and Ag2 are each coordinated to two nitrogen atoms from the thiazolidinone ring of two different ligands (N24 and N34, or N14 and N44, respectively). Accordingly, these two Ag^+^ ions have a coordination number of 2, with a distorted linear geometry, the N–Ag–N angle being closest to 180° in Ag2 (Table 3).

The Ag3 atom is tetracoordinated, binding to four nitrogen atoms—two from the pyridine ring and imine group of a single ligand (N31 and N32), and two others from the hydrazone group of two different ligands (N13 and N43). Calculations using the SHAPE program^34^ (Table S8) show that the geometry around Ag3 is a distorted axially vacant trigonal bipyramid (Figure 2b), with C_3v_ symmetry.

Ag4 is bonded to two N-atoms from hydrazone moieties of two different ligands (N23 and N33). In addition, the Ag4–S4 distance of 2.867(3) Å could seem quite long, and be considered as a secondary interaction rather than a true dative covalent bond. However, though the average Ag–S bond length in three-coordinated Ag^+^ complexes is *ca*. 2.5 Å, these distances can vary from 2.39 to 3.0 Å.^38^ Besides, the N–Ag–S angles, which could seem quite acute, are also in the range of those reported for true Ag–S coordination bonds.^39,40^ Accordingly, Ag4 is also bonded to one S-atom of the thiazolidinone residue of a third ligand (S4) and, therefore, tricoordinated. The geometry around this metal center is distorted mer-octahedral with three vacant positions (T-Shape, Figure 2b) according to SHAPE (Table S8), showing C_2v_ symmetry. All the bond distances and angles in **3** fall within the expected range^24,39,40^ and do not merit further discussion.

In agreement with the above description, the thiazolidinone ring of two of the ligands acts as a κ^1^N donor while for the other two it behaves as a μ_2_-κ^1^N:κ^1^S bridge (Scheme 2). It should be noted that, to the best of our knowledge, this latter coordination mode of the thiazolidinone ring has only been reported for three *Ag*_8_ complexes^24^ (Table 1). In the few known complexes where this ring acts as a bridge, it mainly coordinates through N- and O-atoms (Table 1, Scheme 2d),^9,11^ but not through sulfur. Therefore, this compound highlights the remarkable versatility of this thiazolidinone heterocycle, and its ability to use its three donor atoms in a wide variety of coordination modes.

Besides, in **3**·3DMF, all the deprotonated Am4DHotaz^–^ ligands act as bridges between the four Ag atoms, but exhibit different coordination modes (Table 4, Scheme 5). Two ligands (1 and 2) act as bidentate μ_2_-κ^1^N”:κ^1^N”’ bridges (Scheme 5c). A third ligand (3) behaves as a tetradentate bridge, simultaneously coordinating Ag1, Ag3, and Ag4 through all its nitrogen atoms, resulting in a μ_3_-κ^2^NN’:κ^1^N”:κ^1^N”’ coordination mode (Scheme 5g). The fourth ligand acts as a bidentate bridge through the nitrogen atom N44 and the sulfur atom S4 of the thiazolidinone residue, with a μ_2_-κ^1^N”’:κ^1^S coordination mode (Scheme 5d). It is worth highlighting that none of these three coordination modes have been previously reported for complexes of Am4Rotaz ligands (R = DH, E, or M, Scheme 2 and Scheme of Table S1) or their RTone (Scheme 2) analogues (see Table 4 and Scheme 5). Therefore, this work significantly expands the known coordination behavior of this class of ligands containing thiazolidinone rings.

**Table 4:** Crystallographically Characterized Complexes Derived of Am4Rotaz or RTone Ligands

compound^a^	coord. mode ligand^c^	ref
**κ** ^**1**^ **N-thiazolidinone** ^b^		
[Pd^II^(Am4DHotaz)Cl]	κ^3^NN′N‴	^15^
[Pt^II^(Tone)Cl]	κ^3^NN′N‴	^18,20^
[Cu^II^(Tone)Cl]	κ^3^NN′N‴	^18^
[Fe^III^(NTone)_2_]Cl	κ^3^NN′N‴	^19^
[Fe^III^(Tone)_2_](Fe^III^Cl_4_)	κ^3^NN′N‴	^19^
[Cu^II^(Am4DHotaz)(H_2_O)_2_](ClO_4_)	κ^3^NN′N‴	^22^
[Cu^II^(Am4DHotaz)(NO_3_)]	κ^3^NN′N‴	^22^
**free thiazolidinone** ^b^		
[Pt^II^(Am4Motaz)Cl_2_]	κ^2^NN′	^15^
[Pt^II^(ETone)Cl_2_]	κ^2^NN′	^18,20^
[Zn^II^(HAm4DHotaz)Cl_2_]	κ^2^NN′’	^16^
[Zn^II^(Am4Motaz)Cl_2_]	κ^2^NN’	^16^
[Zn^II^(Am4Eotaz)Cl_2_]	κ^2^NN′	^16^
[Cu^II^(Am4Motaz)_2_Cl]Cl·	κ^2^NN′	^23^
[Fe^III^(Am4Motaz)_2_Cl_2_]Cl	κ^2^NN′	^23^
[Fe^III^(Etone-H)(ETone)_2_](Fe^III^Cl_4_)_2_ ^d^	κ^2^NN′	^19^
[Cu^II^(Am4Motaz)_2_(H_2_O)](ClO_4_)_2_	κ^2^NN′	^22^
[Cu^II^(Am4Motaz)_2_(NO_3_)](NO_3_)	κ^2^NN′	^22^
[Cu^II^(Am4Eotaz)_2_(ClO_4_)](ClO_4_)	κ^2^NN′	^22^
[Cu^II^(Am4Eotaz)_2_(NO_3_)]_3_(NO_3_)_3_	κ^2^NN′	^22^
[Ag^I^(HAm4DHotaz)_2_](NO_3_)	κ^2^NN′	this work
[Ag^I^(Am4Eotaz)_2_](NO_3_)	κ^2^NN′	this work
**κ** ^**1**^ **-N and μ**_**2**_ **-κ** ^**1**^ **N:κ** ^**1**^ **S—thiazolidinone** ^b^		
[Ag^I^_8_(Am4DHotaz)_4_(NO_3_)_3_(H_2_O)(MeOH)](NO_3_)	μ_4_-κ^2^N,N′:κ^1^N″:κ^1^N‴:κ^1^S; μ_4_-κ^1^N:κ^1^N′:κ^1^N″:κ^1^N‴	^24^
{[Ag^I^_8_(Am4DHotaz)_4_(NO_3_)_3_(H_2_O)_2_](NO_3_)}_n_	μ_4_-κ^2^N,N′:κ^1^N″:κ^1^N‴:κ^1^S; μ_4_-κ^1^N:κ^1^N′:κ^1^N″:κ^1^N‴	^24^
{[Ag^I^_8_(Am4DHotaz)_4_(NO_3_)_2_(H_2_O)](NO_3_)(OH)}_n_	μ_4_-κ^2^N,N′:κ^1^N″:κ^1^N‴:κ^1^S; μ_4_-κ^1^N:κ^1^N′:κ^1^N″:κ^1^N‴	^24^
{[Ag^I^_4_(Am4DHotaz)_4_]·3DMF}_n_	μ_2_-κ^1^N″:κ^1^N‴; μ_3‑_κ^2^N:κN′:κ^1^N″:κ^1^N‴; μ_2_- κ^1^N‴:κ^1^S	this work
**κ** ^**1**^ **-S, μ**_**2**_ **-κ** ^**1**^ **N:κ** ^**1**^ **S and μ**_**2**_ **-κ** ^**1**^ **S:κ** ^**1**^ **S—thiazolidinone** ^b^		
[Ag^I^_4_(Am4Eotaz)_4_(NO_3_)_2_(H_2_O)](NO_3_)_2_	μ_2_-κ^3^NN′S:κ^1^S; μ_2_-κ^2^NN′:κ^1^S	this work

a Solvates are omitted.

b Coordination mode of the thiazolidinone residue (Scheme 2).

c Coordination mode of the Am4Rotaz or RTone ligands in Scheme 5.

d Authors claim deprotonation of ETone, to balance the Fe^III^ charge, but do not identify the lost proton.

Finally, as previously mentioned, the tetranuclear units are connected through argentophilic interactions (Figure 2b), giving rise to neutral 1D chains. These chains are further stabilized by hydrogen bonds, primarily between the nitrogen atoms of the NH_2_ groups and the oxygen atoms of thiazolidinone residues from adjacent ligands, thereby contributing to the overall crystal packing of the chain (Figure 2c, Table S9).

#### [Ag_4_(Am4Eotaz)_4_(NO_3_)_2_(H_2_O)]­(NO_3_)_2_·1.18H_2_O (**4**·1.18H_2_O)

This is an ionic tetranuclear complex, and its unit cell is composed of [Ag_4_(Am4Eotaz)_4_(NO_3_)_2_(H_2_O)]^2+^ cations, nitrate anions and water as solvate. A vision of the tetranuclear cation is shown in Figure 3, and its main bond distances and angles are summarized in Table 5.

**Table 5:** Main Distances (Å) and Angles (°) for **4**·1.18H_2_O

Ag1–O11	2.274(2)	Ag3–N31	2.310(3)
Ag1–N11	2.302(3)	Ag3–N32	2.434(2)
Ag1–N12	2.337(3)	Ag3–S2	2.5514(8)
Ag1–S1	2.9312(7)	Ag3–S4	2.6217(8)
Ag2–N21	2.310(3)	Ag3–S3	3.0083(8)
Ag2–N22	2.353(3)	Ag4–O5	2.270(2)
Ag2–O21	2.489(3)	Ag4–N41	2.339(3)
Ag2–O22	2.692(3)	Ag4–N42	2.343(3)
Ag2–S1	2.5802(8)	Ag4–S3	2.6923(8)
Ag2–S2	2.9205(8)	Ag4···S4	3.0997(8)
Ag1···Ag2	3.4491(4)	Ag3···Ag4	3.8871(4)
Ag2···Ag3	3.9846(4)		
O11–Ag1–N11	139.56(8)	S2–Ag3–S4	132.02(3)
O11–Ag1–N12	144.68(8)	N31–Ag3–S3	127.76(6)
N11–Ag1–N12	71.80(8)	N32–Ag3–S4	111.34(6)
O11–Ag1–S1	77.80(6)	N32–Ag3–S3	61.74(6)
N12–Ag1–S1	67.22(6)	O5–Ag4–N41	130.11(9)
N21–Ag2–O21	134.85(8)	O5–Ag4–N42	130.05(8)
N21–Ag2–S1	111.17(7)	O5–Ag4–S3	95.16(6)
N22–Ag2–S1	117.67(6)	N41–Ag4–N42	71.54(9)
N21–Ag2–S2	126.01(7)	N41–Ag4–S3	112.24(7)
N22–Ag2–O21	135.81(8)	N42–Ag4–S3	119.00(6)
N22–Ag2–S2	64.88(6)	Ag1–S1–Ag2	77.19(2)
O22–Ag–S1	134.65(6)	Ag2–S2–Ag3	93.23(2)
Ag1···Ag2···Ag3	114.679(8)	Ag3–S3–Ag4	85.79(3)
Ag2···Ag3···Ag4	172.279(9)		

The cation [Ag_4_(Am4Eotaz)_4_(NO_3_)_4_(H_2_O)]^2+^ contains four crystallographically distinct Ag atoms, each coordinated to one of the four neutral Am4Eotaz ligands, three of them using their thiazolidinone sulfur atoms to bridge two adjacent metal centers.

The four metal centers in the cation exhibit distinct coordination environments. Thus, Ag1 has a coordination number of four, binding to the pyridine (N11) and imine nitrogen (N12) atoms, and the thiazolidinone sulfur (S1) atom of a single Am4Eotaz ligand. Its coordination sphere is completed by an oxygen (O11) atom from a nitrate anion, which acts as monodentate. This results in a highly distorted square planar environment, as determined by SHAPE^34^ calculations (Table S10, Figure 3b). The bond angles (Table 5) reflect this distortion.

Ag2 is hexacoordinated, in an *N*_2_ *O*_*2*_ *S*_2_ environment. This is provided by (a) the coordination to a second Am4Eotaz ligand through its pyridine (N21) and imine nitrogen (N22) atoms, and its thiazolidinone sulfur (S2), as in Ag1; (b) a sulfur (S1) atom from the Am4Eotaz ligand joined to Ag1, thus forming a μ-S bridge between Ag1 and Ag2; (c) two oxygen atoms (O21 and O22) from a terminal bidentate nitrate ligand. This latter is asymmetrically coordinated, as the Ag2–O21 bond is significantly shorter than the Ag2–O22 one (Table 5). The Ag2–O22 distance of 2.692(3) Å could seem quite long to be considered as a coordination bond, given that it is longer than the average Ag–O distance of 2.446 Å found in the CCDC database. Nevertheless, distances of 2.7 Å or longer have been reported as true coordination bonds for many Ag^+^ complexes.^24,41–43^ Thus, Ag2 has coordination number 6, and this *N*_2_ *O*_*2*_ *S*_2_ environment leads to a distorted trigonal prism geometry around the metal ion (SHAPE calculations in Table S10). The high distortion of the polyhedron (Figure 3b) is also evidenced by the deviation of the angles from the ideal 60°, 90°, and 180° (Table 5).

Ag3 is pentacoordinated in an *N*_2_ *S*_*3*_ environment provided by pyridine (N31) and imine nitrogen (N32) atoms, and the thiazolidinone sulfur atom (S3) from a third Am4Eotaz ligand, along with sulfur atoms from two adjacent ligands (S2 and S4). Thus, simple μ-S bridges exist between Ag3 and Ag4 and Ag2 and Ag3, as well as between Ag1 and Ag2 (Figure 3a), with Ag–S–Ag angles ranging from 77° to 94°. Notably, this μ-S ring coordination mode (μ_2‑_κ^1^S:κ^1^S, Scheme 2f), to the best of our knowledge, has not been previously reported for thiazolidinone ligands. Consequently, this work introduces the novel coordination mode μ_2‑_κ^1^S:κ^1^S for thiazolidinones.

The *N*_*2*_ *S*_*3*_ environment for Ag3 leads to a square pyramid geometry, highly distorted toward a trigonal bipyramid (Figure 3b), as SHAPE calculations indicate (Table S10).

Ag4 is also tetracoordinated, as Ag1, but in this case, its coordination environment comprises the pyridine and imine nitrogen atoms of a fourth Am4Eotaz ligand, the sulfur atom S3 from a different ligand, and a water molecule. A second sulfur atom (S4) is located at 3.099 Å, which suggests a secondary interaction, as it exceeds the 3.01 Å threshold generally accepted for Ag–S bonds in tetracoordinated environments.^24,38,44^ Therefore, the geometry around Ag4 is best described as a tetrahedron but highly distorted toward an axially vacant trigonal bipyramid (Figure 3b), in agreement with SHAPE analysis (Table S10). The distortion is supported by the bond angles, ranging from 71.54(9)° to 130.10(9)°.

In this structure, the sulfur-bridged silver atoms are arranged in an L-shaped configuration, as evidenced by the slightly distorted Ag1···Ag2···Ag3 angle of 114.679(8)° and the Ag2···Ag3···Ag4 angle of 172.279(9)°. The sulfur bridges result in Ag···Ag distances ranging from 3.4491(4) to 3.9847(4) Å. These Ag···Ag distances exceed the sum of the van der Waals radii (3.44 Å), indicating negligible or very weak metal–metal interactions. Weak interactions are also shown among the nitrate counterions, water, NH_2_ groups and some carbonyl moieties, which are implicated in hydrogen bonds, giving rise to a 3D supramolecular framework (Table S11).

Furthermore, for three of the four ligands in this complex, the coordination mode is μ_2_-κ^3^NN′S:κ^1^S (Scheme 4f), while for the fourth it is μ_2_-κ^2^NN′:κ^1^S (Scheme 5e). These coordination modes have not also been reported before for Am4Rotaz or RTone ligands. Accordingly, this work introduces five new coordination modes for this type of donor (Scheme 5c–g), highlighting its remarkable versatility. This finding constitutes a significant advance in the coordination chemistry of these ligands, for which only four coordination modes had been reported to date. Notably, this contribution is particularly relevant given that the studies conducted so far on the biological activity of thiazolidinone-containing complexes with promising results have been carried out exclusively with compounds featuring RTone or Am4Rotaz ligands.^18,19,22,23^

## Conclusions

This work presents a new, straightforward, microwave-assisted synthetic method for the isolation of two thiazolidinone-containing Am4Rotaz donors (HAm4DHotaz and Am4Eotaz), which significantly reduces reaction times and enhances both yield and purity compared to reported conventional methods. Furthermore, the coordination chemistry of these donors with silver­(I) nitrate and acetate was investigated, resulting in the isolation of two mononuclear ([Ag­(HAm4DHotaz)_2_]­(NO_3_)·H_2_O and [Ag­(Am4Eotaz)_2_]­(NO_3_)) and two tetranuclear ( [Ag_4_(Am4DHotaz)_4_]·8H_2_O and [Ag_4_(Am4Eotaz)_4_(NO_3_)_2_(H_2_O)]­(NO_3_)_2_·1.18H_2_O) complexes. The findings reveal that Ag:ligand molar relations affect the stoichiometry of the complexes, although HAm4DHotaz and Am4Eotaz do not show the same pattern. In addition, the nature of the anionic counterion also influences the stoichiometry of the resulting complexes for HAm4DHotaz. Thus, its interaction with a neutral salt, as silver nitrate, leads to non-deprotonation of the NH group, and the thiazolidinone residue remains uncoordinated. However, if this reaction is performed with a basic salt, as silver acetate, the NH group deprotonates and the thiazolidinone residue coordinates to the metal ion. Besides, substitution at the nitrogen atom of the thiazolidinone ring also hinders its ability to coordinate, and, in this case, this type of ligand also acts as a κ^2^NN’ donor. Notably, the crystal structures of the tetranuclear complexes reveal a previously unreported coordination mode for the thiazolidinone ring: μ_2_-κ^1^S:κ^1^S. Moreover, five novel coordination modes were identified for the Am4Rotaz donors (μ_2_-κ^1^N″:κ^1^N‴, μ_2_- κ^1^N‴:κ^1^S, μ_2_-κ^2^NN′:κ^1^S, μ_2_-κ^3^NN′S:κ^1^S and μ_3‑_κ^2^NN′:κ^1^N″:κ^1^N‴), which had not also been described before for its RHTone analogues.

Taken together, this work makes a significant contribution to the coordination chemistry of thiazolidinones and their Am4Rotaz or RHtone derivatives, ligands that, to date, represent the only examples within metal complexes of thiazolidinones to exhibit promising biological activity.

## Supplementary Material



## Notes

ESI^+^ mass spectra; IR spectra; ^1^H NMR spectra; molecular structures; crystal data and structure refinement; main bond distances; X-ray tables; and SHAPE calculations (PDF)
