# Supplementary material for: Emerging Trends in the Coordination Chemistry of Thiazolidinone-Containing Polydentate Ligands: Unconventional Binding Modes in Silver Complexes
Source: Inorg Chem. 2025 Jun 27;64(28):14475–86. doi: 10.1021/acs.inorgchem.5c01815 (PMC12284851; doi:10.1021/acs.inorgchem.5c01815)
Supplement: Supplementary file 1 [file ic5c01815_si_001.pdf]

Supporting Information

Emerging Trends in the Coordination Chemistry of Thiazolidinone-containing Polydentate Ligands: Unconventional Binding Modes in Silver Complexes

Julio Corredoira-Vázquez[£,ε] *Manuel Saa,*[§] *Isabel García-Santos,*[§,*] *Alfonso Castiñeiras*[§,] *and Matilde Fondo*[£,*]

[£] Departamento de Química Inorgánica, Facultade de Química, Universidade de Santiago de Compostela, Campus Vida, 15782 Santiago de Compostela, Spain.

[§] Departamento de Química Inorgánica, Facultade de Farmacia, Universidade de Santiago de Compostela, Campus Vida, 15782 Santiago de Compostela, Spain.

[ε] Institute of Materials (iMATUS), Universidade de Santiago de Compostela, 15782 Santiago de Compostela, Spain.

Corresponding authors' e-mail addresses:

Isabel García-Santos, E-mail: isabel.garcia@usc.es

Matilde Fondo, E-mail: matilde.fondo@usc.es

Table S1. Crystallographically characterized complexes bearing non-coordinated thiazolidinone residues. page S3

Figure S1. ESI^+ mass spectra for $[Ag(HAm4DHotaz)_2](NO_3)\cdot H_2O$ (a), $[Ag(Am4Eotaz)_2](NO_3)$ (b), $[Ag_4(Am4DHotaz)_4]\cdot 8H_2O$ (c) and $[Ag_4(Am4Eotaz)_4(NO_3)_2(H_2O)](NO_3)_2\cdot 1.18H_2O$ (d). page S4

Figure S2. IR spectra for $[Ag(HAm4DHotaz)_2](NO_3)\cdot H_2O$ (a), $[Ag(Am4Eotaz)_2](NO_3)$ (b), $[Ag_4(Am4DHotaz)_4]\cdot 8H_2O$ (c) and $[Ag_4(Am4Eotaz)_4(NO_3)_2(H_2O)](NO_3)_2\cdot 1.18H_2O$ (d). page S5

Figure S3. ^{1}H NMR spectra for [Ag(HAm4DHotaz)$_2$](NO$_3$)·H$_2$O (a), [Ag(Am4Eotaz)$_2$](NO$_3$) (b), [Ag$_4$(Am4DHotaz)$_4$]·8H$_2$O (c) and [Ag$_4$(Am4Eotaz)$_4$(NO$_3$)$_2$(H$_2$O)](NO$_3$)$_2$·1.18H$_2$O (d). page S7

Table S2. Crystal data and structure refinement for Am4Eotaz and the side products. page S9

Table S3. Crystal data and structure refinement for silver complexes. page S10

Figure S4. Molecular structure for H124taztAc. page S11

Table S4. Main bond distances [Å] and angles for H124taztAc, including the hydrogen bonds. page S11

Figure S5. Molecular structures for: a) (H$_2$Am4DHotaz)Cl·2H$_2$O; b) (H$_3$Am4DHotaz)Cl$_2$; c) Am4Eotaz. page S12

Table S5. Main bond distances [Å] and angles [º] for (H$_2$Am4DHotaz)Cl·2H$_2$O, (H$_3$Am4DHotaz)Cl$_2$ and Am4EHotaz. page S13

Table S6. Classical hydrogen bonds [Å,º] for (H$_2$Am4DHotaz)Cl·2H$_2$O, (H$_3$Am4DHotaz)Cl$_2$ and Am4Eotaz. page S42

Figure S6. Molecular structure for [Ag(HAm4DHotaz)$_2$](NO$_3$)·H$_2$O (**1**·H$_2$O). page S15

Table S7 SHAPE v2.1. Continuous Shape Measures Calculation (c) 2013, Electronic Structure Group, Universitat de Barcelona, for [Ag(HAm4DHotaz)$_2$](NO$_3$)·H$_2$O (**1**·H$_2$O) and [Ag(Am4Eotaz)$_2$](NO$_3$) (**2**). page S15

Table S8. SHAPE v2.1. Continuous Shape Measures Calculation (c) 2013, Electronic Structure Group, Universitat de Barcelona for [Ag$_4$(HAmDHotaz)$_4$]·3DMF (**3**·3DMF). page S16

Table S9. Hydrogen bonds (Å,°) for [Ag$_4$(HAmDHotaz)$_4$]·3DMF (**3**·3DMF). page S16

Table S10. SHAPE v2.1. Continuous Shape Measures Calculation (c) 2013, Electronic Structure Group, Universitat de Barcelona, for [Ag$_4$(Am4Eotaz)$_4$(NO$_3$)$_2$(H$_2$O)](NO$_3$)$_2$·1.18H$_2$O (**4**·1.18H$_2$O). page S17

Table S11. Hydrogen bonds (Å,°) for [Ag$_4$(Am4Eotaz)$_4$(NO$_3$)$_2$(H$_2$O)](NO$_3$)$_2$·1.18H$_2$O. (**4**·1.18H$_2$O). page S18

References page S19

Table S1. Crystallographically characterized complexes bearing non-coordinated thiazolidinone residues.

Compound*	Ref.
[Cu(napt)Cl]	S1
[Pt(Am4Motaz)Cl_2]	S2
[Pt(ETone)Cl_2]	S3, S4
[Zn(HAm4DHotaz)Cl_2]	S5
[Zn(Am4Motaz)Cl_2]	S5
[Zn(Am4Eotaz)Cl_2]	S5
[Cu(Am4Motaz)$_2$Cl]Cl·	S6
[Fe(Am4Motaz)$_2$$Cl_2$]Cl	S6
[Fe(Etone-H)(ETone)$_2$]($FeCl_4$)$_2$[a]	S7
[Cu(Am4Motaz)$_2$(H_2O)](ClO_4)$_2$	S8
[Cu(Am4Motaz)$_2$(NO_3)](NO_3)	S8
[Cu(Am4Eotaz)$_2$(ClO_4)](ClO_4)	S8
[Cu(Am4Eotaz)$_2$(NO_3)]$_3$(NO_3)$_3$	S8
[Ag(HAm4DHotaz)$_2$](NO_3)	This work
[Ag(Am4Eotaz)$_2$](NO_3)	This work

* Solvates are omitted, ligands in Scheme below; [a] authors claim deprotonation of ETone, to balance the Fe^{III} charge, but do not identify the lost proton.

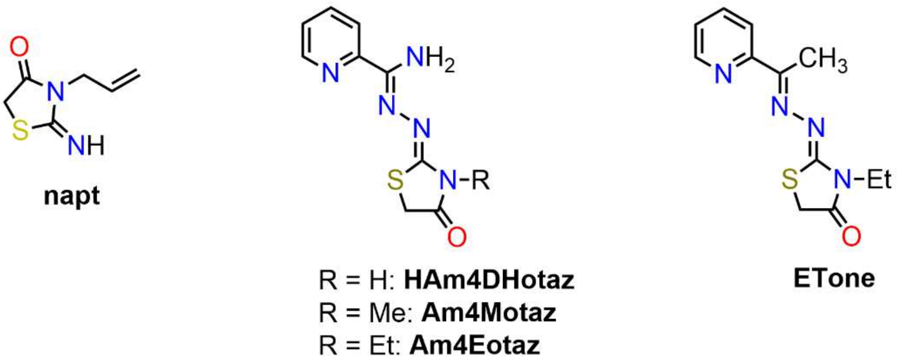

Ligands in Table S1

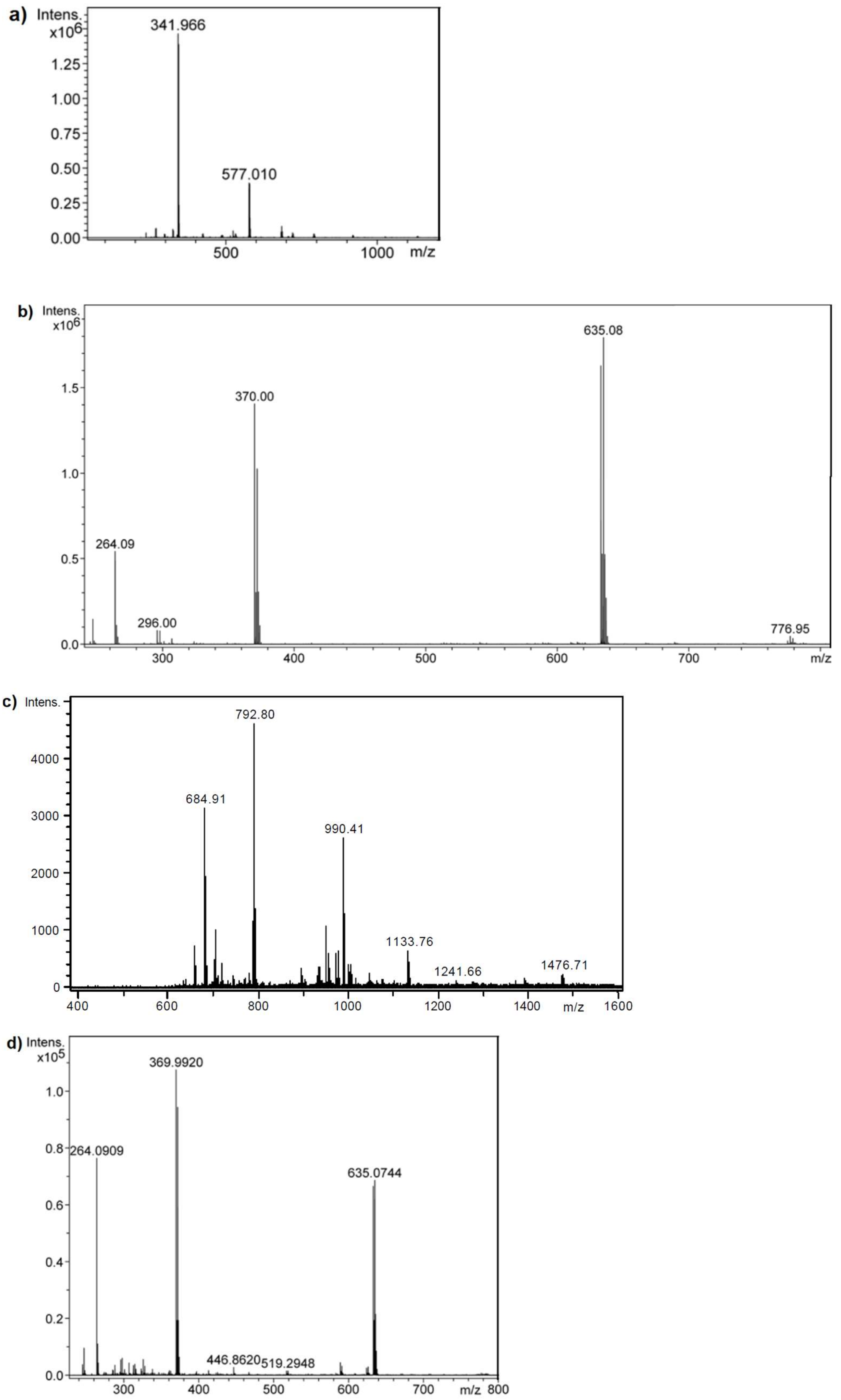

Figure S1. ESI^+ mass spectra for $[Ag(HAm4DHotaz)_2](NO_3)\cdot H_2O$ (a), $[Ag(Am4Eotaz)_2](NO_3)$ (b), $[Ag_4(Am4DHotaz)_4]\cdot 8H_2O$ (c) and $[Ag_4(Am4Eotaz)_4(NO_3)_2(H_2O)](NO_3)_2\cdot 1.18H_2O$ (d).

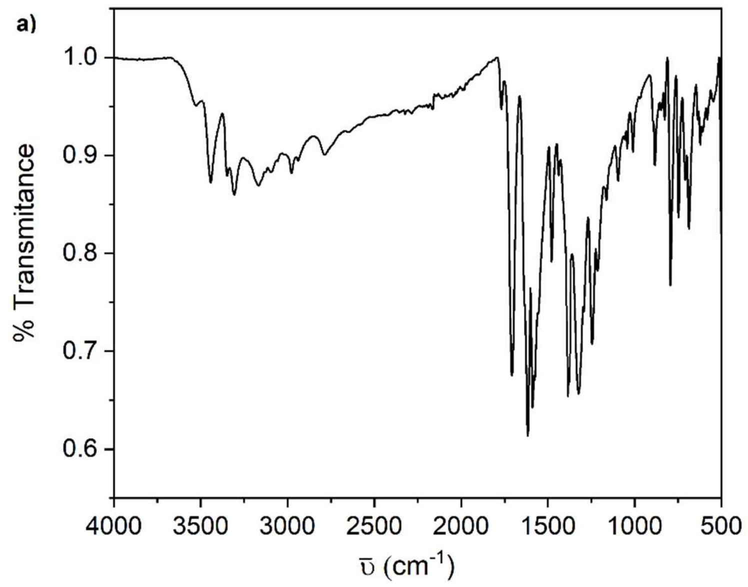
a)
% Transmitance
1.0
0.9
0.8
0.7
0.6
4000
3500
3000
2500
2000
1500
1000
500
ῡ (cm-1)

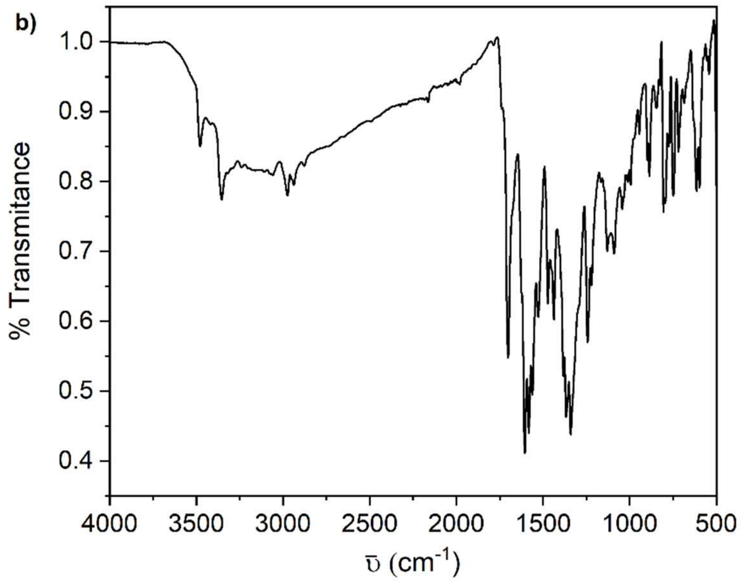
b)
% Transmitance
1.0
0.9
0.8
0.7
0.6
0.5
0.4
4000
3500
3000
2500
2000
1500
1000
500
ῡ (cm-1)

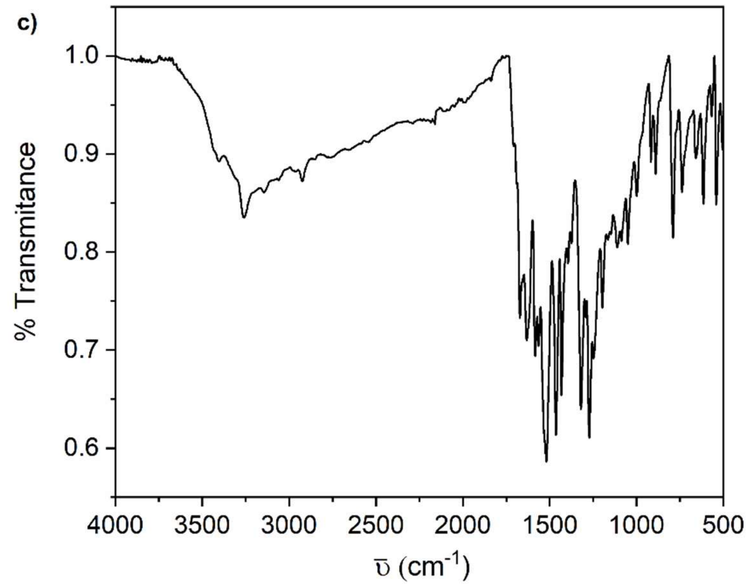
c)
% Transmitance
1.0
0.9
0.8
0.7
0.6
4000
3500
3000
2500
2000
1500
1000
500
ῡ (cm-1)

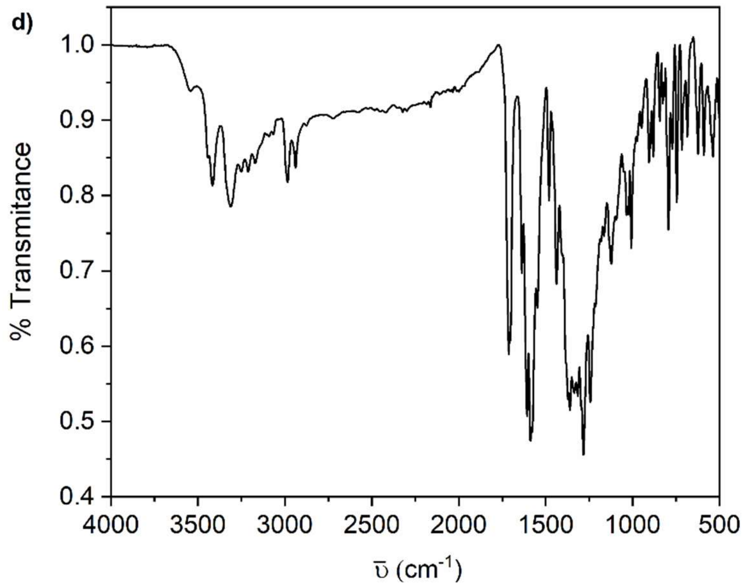

Figure S2. IR spectra for $[Ag(HAm4DHotaz)_2](NO_3)\cdot H_2O$ (a), $[Ag(Am4Eotaz)_2](NO_3)$ (b), $[Ag_4(Am4DHotaz)_4]\cdot 8H_2O$ (c) and $[Ag_4(Am4Eotaz)_4(NO_3)_2(H_2O)](NO_3)_2\cdot 1.18H_2O$ (d) in the range 500-4000 cm^{-1}.

a)

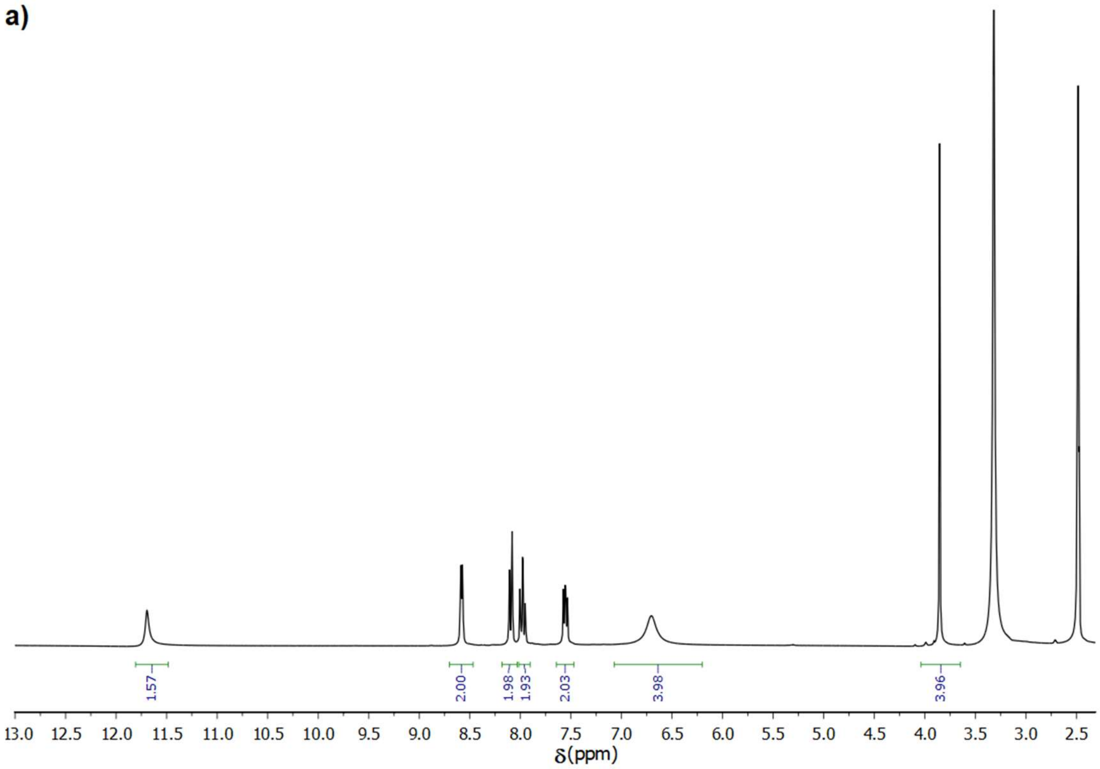
1.57
2.00
1.98
1.93
2.03
3.98
3.96
13.0 12.5 12.0 11.5 11.0 10.5 10.0 9.5 9.0 8.5 8.0 7.5 7.0 6.5 6.0 5.5 5.0 4.5 4.0 3.5 3.0 2.5
δ(ppm)

b)

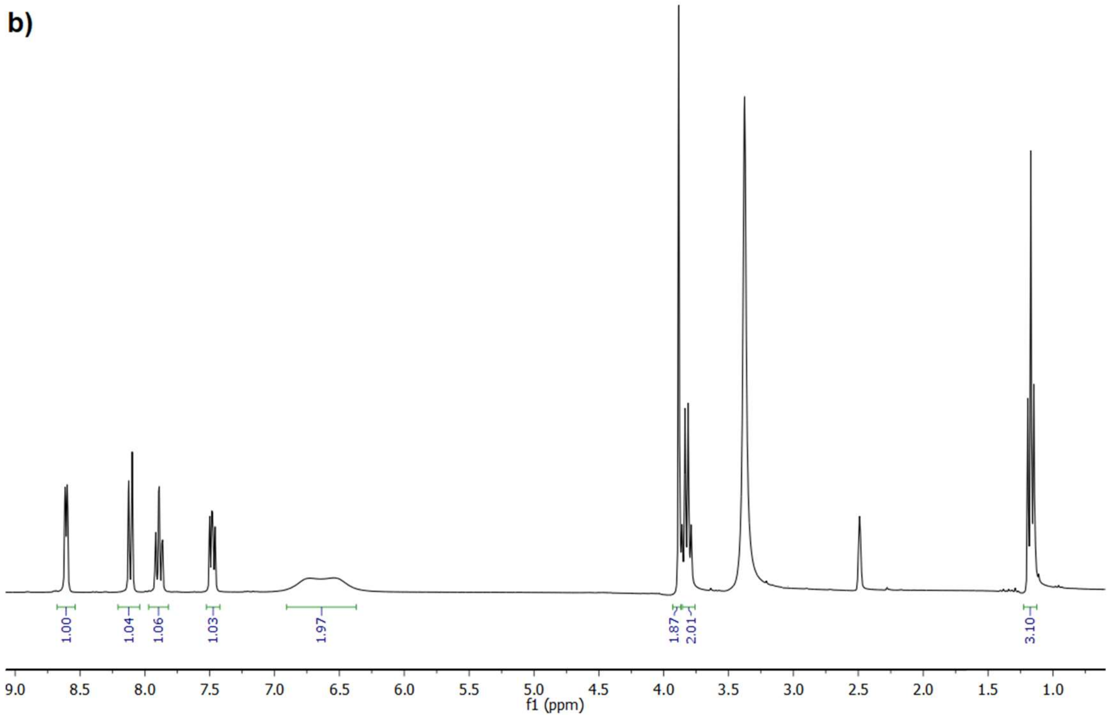
1.00
1.04
1.06
1.03
1.97
1.87
2.01
3.10
9.0 8.5 8.0 7.5 7.0 6.5 6.0 5.5 5.0 4.5 4.0 3.5 3.0 2.5 2.0 1.5 1.0
f1 (ppm)

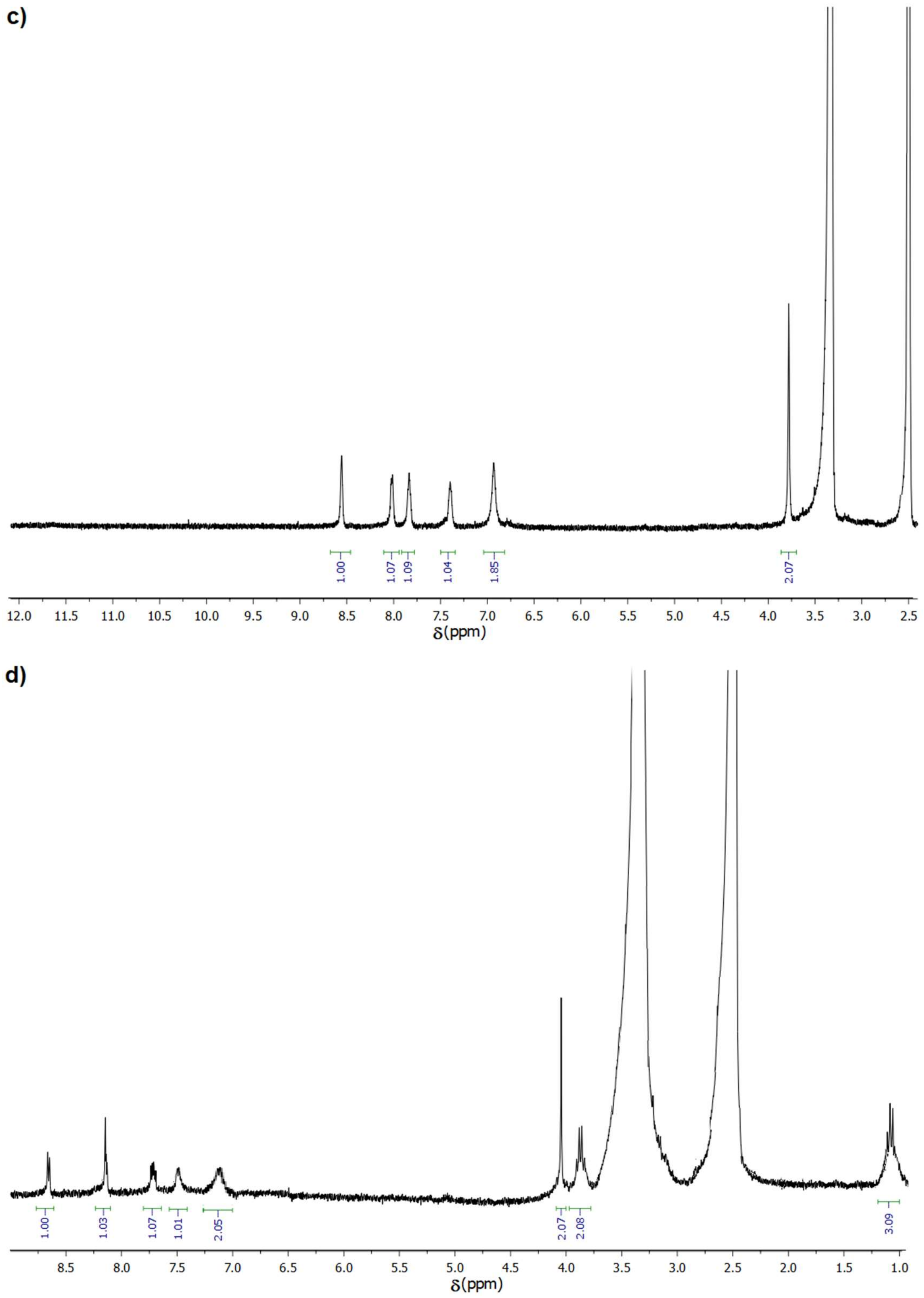

Figure S3. ^{1}H NMR spectra in DMSO-d$_6$ for [Ag(HAm4DHotaz)$_2$](NO$_3$)·H$_2$O between 2.5-13 ppm (a), [Ag(Am4Eotaz)$_2$](NO$_3$) between 0.5-9 ppm (b), [Ag$_4$(Am4DHotaz)$_4$]·8H$_2$O between 2.5-12 ppm (c) and [Ag$_4$(Am4Eotaz)$_4$(NO$_3$)$_2$(H$_2$O)](NO$_3$)$_2$·1.18H$_2$O between 1-9 ppm (d).

Table S2. Crystal data and structure refinement for Am4Eotaz and the side products.

	H124taztAc	$(H_2Am4DHotaz)Cl\cdot 2H_2O$	$(H3Am4DHotaz)Cl_2$	Am4Eotaz
Empirical formula	$C_9H_8N_4O_2S$	$C_9H_{14}ClN_5O_3S$	$C_9H_{11}Cl_2N_5OS$	$C_{11}H_{13}N_5OS$
Formula weight	236.25	307.76	308.19	263.32
Temperature/K	293(2)	100(2)	100(2)	100(2)
Wavelength/Å	0.71073	0.71073	0.71073	0.71073
Crystal system	Monoclinic	Triclinic	Monoclinic	Monoclinic
Space group	$P2_1/c$	$P\bar{1}$	$P2_1/n$	$P2_1/n$
a/Å	4.9288(5)	7.83330(10)	5.0694(2)	10.4141(5)
b/Å	14.9283(15)	8.77520(10)	18.9380(8)	8.9862(4)
c/Å	13.5497(14)	10.98110(10)	13.6018(6)	13.7308(6)
α/º	90	105.5110(10)	90	90
β/º	90.286(6)	91.3500(10)	92.743(3)	104.609(2)
γ/º	90	112.6310(10)	90	90
Volume/$Å^{-3}$	996.96(18)	664.511(14)	1304.34(9)	1243.43(10)
Z	4	2	4	4
Calc. density/g/m^3	1.574	1.538	1.569	1.407
Absorp. coefc./mm^{-1}	2.842	0.457	0.653	0.256
Crystal size/mm	0.10x0.08x0.04	0.32x0.30x0.14	0.32x 0.02 x 0.02	0.43x0.17x0.10
θ range/º	4.407-65.265	1.944-32.574	1.845-26.510	2.21-36.13
Refl. collect/unique	11003/1695	20771/4827	24081/2678	28354/5937
Completeness to theta θ /%	65.27/99.5	25.242/99.9	25.242/100	36.13/99.9
Max./min. transm.	1.000/0.854	1.000/0.862	1.000/0.743	1.000/0.9209
Data/parameters	1695/146	4827/172	2678/163	5937/163
Goodness-of-fit on F^2	1.067	1.033	0.934	1.174
Final *R* indices	R_1=0.0751 wR_2=0.1883	R_1=0.0233 wR_2=0.0649	R_1=0.0519 wR_2=0.1014	R_1=0.0575 wR_2=0.1158
R indices (all data)	R_1=0.0846 wR_2=0.1972	R_1=0.0256 wR_2=0.0661	R_1=0.1069 wR_2=0.1161	R_1=0.0666 wR_2=0.1194

Table S3. Crystal data and structure refinement for silver complexes.

	**1**·H_2O	**2**	**3**·3DMF	**4**·1.18H_2O
Empirical formula	$C_{18}H_{20}AgN_{11}O_6S_2$	$C_{22}H_{26}AgN_{11}O_5 S_2$	$C_{45}H_{53}Ag_4N_{23}O_7S_4$	$C_{44}H_{56.37}Ag_4N_{24}O_{18.18}S_4$
Formula weight	658.44	696.53	1587.82	1772.18
Temperature/K	100(2)	100(2)	100(2)	100(2)
Wavelength/Å	0.71073	0.71073	0.71073	0.71073
Crystal system	Monoclinic	Triclinic	Monoclinic	Monoclinic
Space group	$P2_1/n$	$P\bar{1}$	$P2_1/c$	$P2_1/c$
a/Å	7.3579(3)	11.0414(2)	11.6171(9)	11.0352(2)
b/Å	16.8954(6)	11.0577(2)	35.654(2)	52.5797(12)
c/Å	19.4648(8)	13.0788(3)	14.1260(10)	11.8075(3)
α/º	90	98.8350(10)	90	90
β/º	90.067(2)	103.9930(10)	101.708(3)	115.9890(10)
γ/º	90	111.6480(10)	90	90
Volume/$Å^{-3}$	2419.76(16)	1386.99(5)	5729.2(7)	6158.2(2)
Z	4	2	4	4
Calc. density/mg/m^3	1.807	1.668	1.841	1.911
Absorp. coefc./mm^{-1}	1.067	0.933	1.564	1.479
Crystal size/mm	0.48x0.10x0.10	0.30x0.27x0.02	0.12x0.07x0.02	0.12x0.10x0.06
θ range/º	1.05/28.22	1.67/27.88	1.58/24.73	0.77/27.88
Refl. collect/unique	53009/5959	58520/6589	49672/9791	118662/14664
Completeness to theta θ /%	25.24/100	25.24/99.9	25.24/94.7	25.24/100
Max./min. transm.	1.000/0.6976	1.0000/0.7814	1.0000/0.8609	0.9165/0.8425
Data/parameters	5959/344	6589/370	9791/713	14664/856
Goodness-of-fit on F^2	1.082	1.076	0.950	1.097
Final *R* indices	R1=0.0296 wR2 =0.0619	R1=0.0279 wR2=0.0634	R1 =0.0726 wR2 =0.1110	R1=0.0364 wR2=0.0656
R indices (all data)	R1=0.0345 wR2 =0.0633	R1=0.0377 wR2=0.0660	R1=0.1553 wR2 =0.1317	R1=0.0501 wR2=0.0695

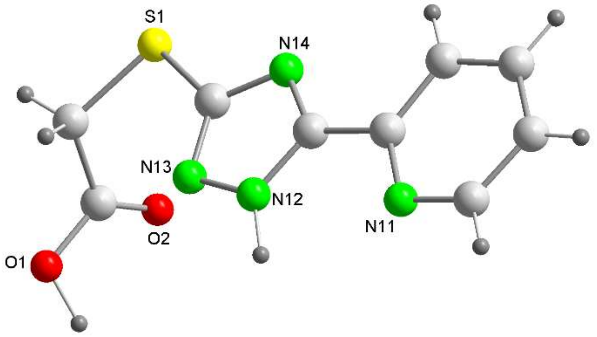

Figure S4. Molecular structure for H124taztAc.

Table S4. Main bond distances [Å] and angles for H124taztAc, including the hydrogen bonds.

S1-C17	1.746(4)	N14-C16	1.335(5)	
N12-N13	1.369(4)	S-C1	1.798(4)	
N12-C16	1.340(5)	C1-C2	1.512(5	
N13-C17	1.326(5)	O1-C2	1.324(5)	
N14-C17	1.359(5)	O2-C2	1.210(4)	
C15-C16	1.459(5)			
C15-N11-C11	116.6(3)	N11-C15-C16	123.2(3)	
C16-N12-N13	110.5(3)	N13-C17-N14	114.4(3)	
C17-N13-N12	102.2(3)	N11-C11-C12	124.2(4)	
N13-C17-S	123.5(3)	C17-S1-C1	98.22(17)	
N14-C17-S	122.0(3)			
D-H…A	d(D-H)	d(H…A)	d(D…A))	<(DHA
O1-H1···N14[1]	1.07	1.91	2.665(4)	124.3
N12-H12A···N11[2]	1.03	2.05	2.954(4)	144.8

[1]x, -y+1/2, z+1/2; [2] -x+1, -y+1, -z+1

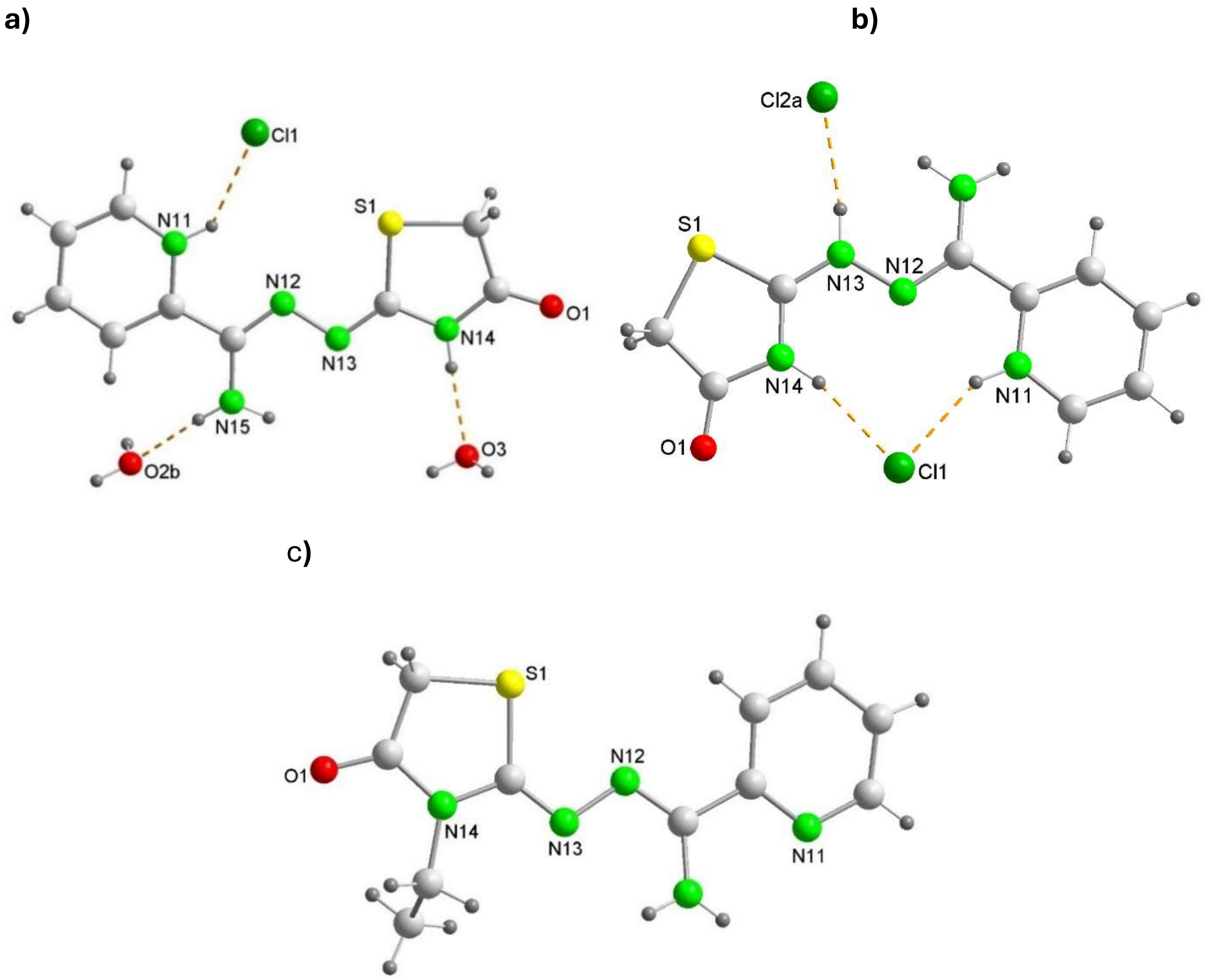

Figure S5. Molecular structures for: a) $(H_2Am4DHotaz)Cl\cdot 2H_2O$; b) $(H_3Am4DHotaz)Cl_2$; c) Am4Eotaz.

Table S5. Main bond distances [Å] and angles [º] for $(H_2Am4DHotaz)Cl\cdot 2H_2O$, $(H_3Am4DHotaz)Cl_2$ and Am4EHotaz.

	$(H_2Am4DHotaz)Cl\cdot 2H_2O$	$(H_3Am4DHotaz)Cl_2$	Am4Eotaz
S-C17	1.7550(7)	1.733(4)	1.7576(12)
S-C18	1.8142(8)	1.810(4)	1.8148(12)
O1-C19	1.2260(9)	1.213(4)	1.2260(14)
N11-C11	1.3414(9)	1.338(5)	1.3425(15)
N11 C15	1.3537(9)	1.349(4)	1.3442(15)
N12-N13	1.4013(8)	1.386(4)	1.4097(13)
N12-C16	1.3021(9)	1.309(4)	1.3029(14)
N13-C17	1.2862(10)	1.313(4)	1.2844(14)
N14-C17	1.3808(9)	1.331(4)	1.3906(14)
N14-C19	1.3534(9)	1.399(4)	1.3668(14)
N15-C16	1.3504(9)	1.328(4)	1.3479(15)
C18-C19	1.5122(10)	1.497(5)	1.5110(17)
C15-C16	1.4805(10)	1.495(5)	1.4884(15)
C19-N14-C17	116.76(6)	115.3(3)	116.01(10)
N12-C16-N15	126.43(7)	128.4(3)	125.82(10)
N12-C16-C15	115.33(6)	113.3(3)	116.74(10
N15-C16-C15	118.24(6)	118.3(3)	117.40(9)
N13-C17-N14	120.73(7)	123.2(3)	122.00(10)
C17-S1-C18	91.27(3)	89.98(17)	91.43(5)
N13-C17-S	127.01(6)	121.5(3)	125.59(8)
N14-C17-S	112.26(5)	115.3(3)	112.40(8)
C19-C18-S	107.48(5)	108.4(2)	107.60(8)
O1-C19-N14	123.70(7)	123.1(4)	123.53(11)
O1-C19-C18	124.13(7)	126.2(3)	124.14(10)

Table S6. Classical hydrogen bonds [Å,º] for (H_2Am4DHotaz)Cl·2H_2O, (H_3Am4DHotaz)Cl_2 and Am4Eotaz.

D-H…A	d(D-H)	d(H…A)	d(D…A)	<(DHA)
**(H_2Am4DHotaz)Cl·2H_2O**				
N11-H11A···Cl1	0.88	2.43	3.1430(7)	138.1
N14-H14A···O3	0.86	1.95	2.8127(9)	174.9
N15-H15A···Cl1[1]	0.87	2.61	3.3502(7)	143.8
N15-H15B···O2[2]	0.84	2.03	2.8563(9)	166.8
O2-H2A···Cl1[1]	0.82	2.36	3.1718(7)	175
O2-H2B···O1[3]	0.81	1.93	2.7307(8)	171.8
O3-H3A···Cl1[4]	0.82	2.39	3.2048(6)	174.2
O3-H3B···Cl1[1]	0.80	2.43	3.2321(6)	174.8
[1] x,y-1,z; [2] -x+1,-y,-z; [3] -x+2,-y+1,-z+1; [4] -x+1,-y+1,-z+1				
**(H_3Am4DHotaz)Cl_2**				
N11-H11A···Cl1	0.88	2.24	3.061(3)	154.0
N13-H13A··Cl2[1]	0.88	2.19	3.065(3)	171.3
N14-H14A···Cl1	0.88	2.24	3.101(3)	166.3
N15-H15B Cl1[1]	0.88	2.29	3.141(3)	162.4
N15-H15B Cl2[1]	0.88	2.32	3.194(3)	172.4
[1] -1/2+x, 1/2-y, -1/2+z				
**AmEotaz**				
N15-H15B···N11	0.84	2.43	2.7200 (14)	100.8
N15-H15A···N13	0.83	2.28	2.6250 (13)	105.3
N15-H15B···O1[1]	0.84	0.84	3.0429 (13)	156.5
[1]x-1/2, -y+1/2, z+1/2				

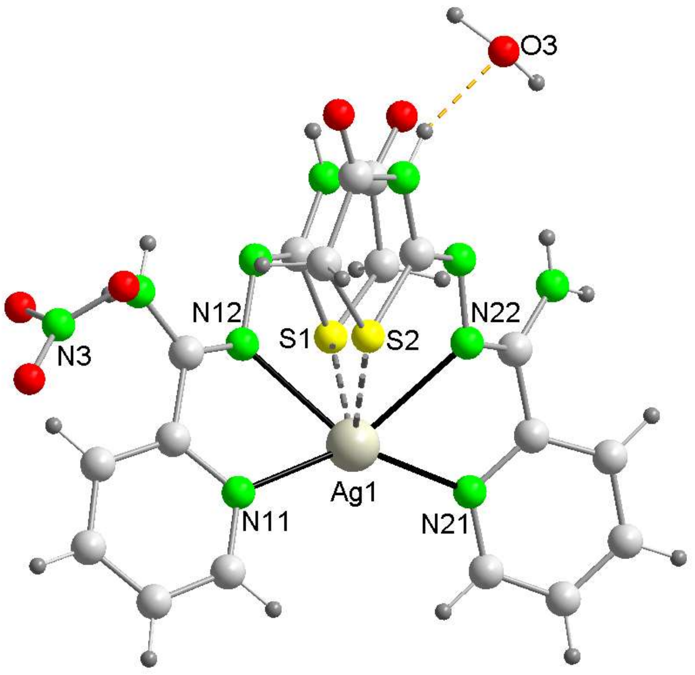

Figure S6. Molecular structure for [Ag(HAm4DHotaz)$_2$](NO$_3$)·H$_2$O (**1**·H$_2$O).

Table S7. SHAPE v2.1. Continuous Shape Measures Calculation (c) 2013, Electronic Structure Group, Universitat de Barcelona, for [Ag(HAm4DHotaz)$_2$](NO$_3$)·H$_2$O (**1**·H$_2$O) and [Ag(Am4Eotaz)$_2$](NO$_3$) (**2**).

**Coordination number 4**		
vTBPY-4	4 C3v	Axially vacant trigonal bipyramid
SS-4	3 C2v	Seesaw or sawhorse (cis-divacant octahedron)
T-4	2 Td	Tetrahedron
SP-4	1 D4h	Square planar

1·H$_2$O

Structure [ML4]	vTBPY-4	**SS-4**	T-4	SP-4
	15.265,	**10.596**,	14.622,	13.187

2

Structure [ML4]	vTBPY-4	**SS-4**	T-4	SP-4
	16.716,	**10.752**,	15.938,	11.625

Table S8. SHAPE v2.1. Continuous Shape Measures Calculation (c) 2013, Electronic Structure Group, Universitat de Barcelona for [$Ag_4(HAmDHotaz)_4$]·3DMF (**3**·3DMF).

**Coordination number 3**		
mvOC-3	4 C2v	mer-Trivacant octahedron
fvOC-3	3 C3v	fac-Trivacant octahedron
vT-3	2 C3v	Vacant tetrahedron
TP-3	1 D3h	Trigonal
**Coordination number 4**		
vTBPY-4	4 C3v	Axially vacant trigonal bipyramid
SS-4	3 C2v	Seesaw or sawhorse (cis-divacant octahedron)
T-4	2 Td	Tetrahedron
SP-4	1 D4h	Square planar

**Ag3**				
Structure [ML4]	**vTBPY-4**	SS-4	T-4	SP-4
	**6.531**,	8.311,	8.232,	23.743
**Ag4**				
Structure [ML3]	**mvOC-3**	fvOC-3	vT-3	TP-3
	**2.857**,	9.898,	4.940,	4.868

Table S9. Hydrogen bonds (Å,°) for [$Ag_4(HAmDHotaz)_4$]·3DMF (**3**·3DMF).

D-H···A	d(D-H)	d(H···A)	d(D···A)	<(DHA)
N15-H15A...O3[1]	0.88	2.27	3.002(11)	141.2
N15-H15B...O7	0.88	2.22	3.018(12)	150.4
N25-H25A...O4[2]	0.88	2.34	2.922(11)	124.2
N25-H25B...N21	0.88	2.36	2.694(13)	103.0
N35-H35A...O4[2]	0.88	2.53	3.221(12)	136.5
N35-H35B...O6	0.88	2.06	2.911(12)	161.8
N45-H45B...N41	0.88	2.34	2.690(14)	104.1

[1] x, -y+1/2, z+1/2; [2] x, -y+1/2, z-1/2

Tabla S10. SHAPE v2.1. Continuous Shape Measures Calculation (c) 2013, Electronic Structure Group, Universitat de Barcelona, for $[Ag_4(Am4Eotaz)_4(NO_3)_2(H_2O)](NO_3)_2 \cdot 1.18H_2O$ (**4**·1.18H_2O).

**Coordination number 4**		
vTBPY-4	4 C3v	Axially vacant trigonal bipyramid
SS-4	3 C2v	Seesaw or sawhorse (cis-divacant octahedron)
T-4	2 Td	Tetrahedron
SP-4	1 D4h	Square planar
**Coordination number 5**		
JTBPY-5	5 D3h	Johnson trigonal bipyramid J12
SPY-5	4 C4v	Spherical square pyramid
TBPY-5	3 D3h	Trigonal bipyramid
vOC-5	2 C4v	Vacant octahedron
PP-5	1 D5h	Pentagon
**Coordination number 6**		
JPPY-6	5 C5v	Johnson pentagonal pyramid J2
TPR-6	4 D3h	Trigonal prism
OC-6	3 Oh	Octahedron
PPY-6	2 C5v	Pentagonal pyramid
HP-6	1 D6h	Hexagon

**Ag1**					
Structure [ML4]	vTBPY-4	SS-4	T-4	**SP-4**	
	28.680,	20.357,	32.113,	**6.160**	
**Ag2**					
Structure [ML6]	JPPY-6	**TPR-6**	OC-6	PPY-6	HP-6
	19.357,	**6.854**,	14.670,	15.805,	29.421
**Ag3**					
Structure [ML5]	JTBPY-5	**SPY-5**	TBPY-5	vOC-5	PP-5
	8.968,	**4.939**,	5.672,	9.572,	35.712
**Ag4**					
Structure [ML4]	vTBPY-4	SS-4	**T-4**	SP-4	
	6.875,	9.713,	**6.011**,	24.552	

Table S11. Hydrogen bonds (Å,°) for $[Ag_4(Am4Eotaz)_4(NO_3)_2(H_2O)](NO_3)_2·1.18H_2O$ (**4**·$1.18H_2O$).

D-H···A	d(D-H)	D(H···A)	D(D···A)	<DHA
O5-H5A...O42	0.87	1.99	2.8484	167.1
O5-H5B...O43[1]	0.79	2.05	2.8203	166.8
N15-H15A...O13[2]	0.81	2.53	3.1974	141.4
N15-H15B...O6[3]	0.83	2.12	2.9373	167.2
N25-H25A...O32	0.92	2.09	2.9774	162.4
N25-H25B...O4[4]	0.85	2.63	3.4334	158.7
N35-H35A...O32	0.89	2.17	3.0274	161.7
N45-H45A...O3[5]	0.81	2.22	2.9473	149.6
N45-H45B...O42[6]	0.85	2.23	2.9964	150.8
N45-H45B...O43[6]	0.85	2.53	3.2794	148.2
O6-H6A...O21	0.77	2.16	2.9183	167.9
O6-H6B...O11	0.78	2.11	2.8893	174.9
O7-H7B...O33[7]	0.83	1.98	2.51014	120.6

[1] x, y+1/2, z+1/2; [2] -x+1, -y, -z+1; [3] x+1, y, z; [4] x, y, z-1; [5] x+1, y, z+1; [6] x+1, -y+1/2, z+1/2; [7] x-1, y, z; [8] 1-x, -y, 2-z

References

S1. Fedorchuk, A. A.; Kinzhybalo, V. V.; Slyvka, Y. I.; Goreshnik, E. A.; Bednarchuk, T. J.; Lis, T.; Myskiv, M. G. Unexpected complexation of allylpseudothiohydantoin hydrochlorides towards CuX (X = Cl, NO_3, ClO_4, BF_4, 1/2SiF_6). The first known examples of joint Cu^I(Cl,ClO_4) and Cu^I(Cl,BF_4) π-complexes, *J. Coord. Chem.* **2017**, *70*, 871-884.

S2. Castiñeiras, A.; García-Santos, I.; Saa, M. Synthesis, structural characterization and properties of the palladium(II) and platinum(II) complexes of 2-{2-[(pyridin-2-yl)aminomethylene]hydrazono}-thiazolidin-4-one and the 3-methyl derivative, *Z. Anorg. Allg. Chem.* **2008**, *634*, 2281-2290.

S3. Song, X-Q.; Liu, Y-H.; Shao, J.; Zhang, Z-L.; Xie, C-Z.; Qiao, X.; Bao, W-G.; Xu, J-Y. Rapid induction of apoptosis in tumor cells treated with a new platinum(II) complex based on amino-thiazolidinone, *Eur. J. Med. Chem.* **2018**, *157*, 188-197.

S4. Shaoa, J.; Weib, J-X.; Zhanga, Y.; Xu, J-Y. Spectroscopic investigations of the interactions of potential antitumor amino-thiazolidinone platinum (II) compounds with human serum albumin, *Inorg. Chem. Commun.* **2019**, *102*, 35-39.

S5. Lin, X.-D.; Peng, B.; Li, S.-Y.; Shao, J.; Li, Q.-Z.; Xie, C.-Z.; Xu, J-Y. Novel Zn(II)-thiazolone-based solid fluorescent chemosensors: naked-eye detection for acid/base and toluene, *RSC Adv*. **2016**, *6*, 52310-52317.

S6. Shao, J.; Zhang, Q.; Wei, J.; Yuchi, Z.; Cao, P.; Li, S-Q.; Wang, S.; Xu, J-Y.; Yang, S.; Zhang, Y.; Wei, J-X.; Tian, J-L. Synthesis, crystal structures, anticancer activities and molecular docking studies of novel thiazolidinone Cu(II) and Fe(III) complexes targeting lysosomes: special emphasis on their binding to DNA/BSA, *Dalton Trans*. **2021**, *50*, 13387-13398.

S7. Wu, Yi-G.; Wang, D-B.; Hu, J-J.; Song, X-Q.; Xie, C-Z.; Ma, Z-Y.; Xu, J-Y. An iron(III) complex selectively mediated cancer cell death: crystal structure, DNA targeting and in vitro antitumor activities, *Inorg. Chem. Front*. **2019**, *6*, 1040-1049.

S8. Alzuet, G.; Castiñeiras, A.; Cores, I.; García-Santos, I.; González-Álvarez, M.; Saa, M. Structural basis and effect of copper(II) complexes with 4-oxo-thiazolidine ligands on DNA binding and nuclease activity, *J. Inorg. Biochem*. **2020**, *203*, 110902.
